# Engineering of thioesterase YciA from *Haemophilus influenzae* for production of carboxylic acids

**DOI:** 10.1007/s00253-023-12691-1

**Published:** 2023-08-12

**Authors:** Laura Pöschel, Mónica Guevara-Martínez, David Hörnström, Antonius J. A. van Maris, Markus Buchhaupt

**Affiliations:** 1 DECHEMA-Forschungsinstitut, Microbial Biotechnology, Theodor-Heuss-Allee 25, 60486 Frankfurt Am Main, Germany; 2 Faculty of Biological Sciences, Goethe University Frankfurt, Max-Von-Laue-Str. 9, 60438 Frankfurt Am Main, Germany; 3 Department of Industrial Biotechnology, School of Engineering Sciences in Chemistry, Biotechnology and Health, KTH Royal Institute of Technology, AlbaNova University Center, SE 10691 Stockholm, Sweden

**Keywords:** *Methylorubrum extorquens*,, Thioesterase,, Dicarboxylic acids,, 3-Hydroxybutyric acid,, Enzyme engineering

## Abstract

**Abstract:**

Acyl-CoA-thioesterases, which hydrolyze acyl-CoA-esters and thereby release the respective acid, have essential functions in cellular metabolism and have also been used to produce valuable compounds in biotechnological processes. Thioesterase YciA originating from *Haemophilus influenzae* has been previously used to produce specific dicarboxylic acids from CoA-bound intermediates of the ethylmalonyl CoA pathway (EMCP) in *Methylorubrum extorquens*. In order to identify variants of the YciA enzyme with the capability to hydrolyze so far inaccessible CoA-esters of the EMCP or with improved productivity, we engineered the substrate-binding region of the enzyme. Screening a small semi-rational mutant library directly in *M.* *extorquens* yielded the F35L variant which showed a drastic product level increase for mesaconic acid (6.4-fold) and 2-methylsuccinic acid (4.4-fold) compared to the unaltered YciA enzyme. Unexpectedly, in vitro enzyme assays using respective *M.* *extorquens* cell extracts or recombinantly produced thioesterases could not deliver congruent data, as the F35L variant showed strongly reduced activity in these experiments. However, applied in an *Escherichia coli* production strain, the protein variant again outperformed the wild-type enzyme by allowing threefold increased 3-hydroxybutyric acid product titers. Saturation mutagenesis of the codon for position 35 led to the identification of another highly efficient YciA variant and enabled structure-function interpretations. Our work describes an important module for dicarboxylic acid production with *M. extorquens* and can guide future thioesterase improvement approaches.

**Key points:**

• *Substitutions at position F35 of YciAHI changed the productivity of YciA-based release of carboxylic acid products in M. extorquens AM1 and E. coli.*

• *YciAHI F35N and F35L are improved variants for dicarboxylic production of 2-methylsuccinic acid and mesaconic acid with M. extorquens AM1.*

• *In vitro enzyme assays did not reveal superior properties of the optimized protein variants.*

**Supplementary Information:**

The online version contains supplementary material available at 10.1007/s00253-023-12691-1.

## Introduction

Thioesterases (EC 3.1.2.1–EC 3.1.2.27) hydrolyze thioesters to the thiol and its carboxylic acid component. They play a central role in basal cellular mechanisms of all living cells in metabolic networks and as regulatory enzymes (Swarbrick et al. 2020). Acyl-CoA thioesterases (ACOTs) are an important target for the engineering of biotechnological production strains. With their critical role in lipid metabolism, modified ACOTs have the potential to vary intracellular levels of acyl-CoAs, free fatty acids, and CoA-SH in the cell (Hunt and Alexson 2002). Moreover, ACOTs have been used as key catalysts for product release of hydroxy acids and carboxylic acids in various microbial production strains (Gao et al. 2002; Lee and Lee 2003; Liu et al. 2007; Martin and Prather 2009; Tseng et al. 2009; Chung et al. 2009; Martin et al. 2013; Sonntag et al. 2014; Guevara-Martínez et al. 2015, 2019; Jarmander et al. 2015; Perez-Zabaleta et al. 2016; Pöschel et al. 2022). For example, acyl-CoA thioesterase YciAEc (EC 3.1.2.20) is used in the production of 3-hydroxybutyric acid (3HB) from (*R*)-3-hydroxybutyryl-CoA with an engineered *Escherichia* *coli* strain (Guevara-Martínez et al. 2015; Jarmander et al. 2015; Perez-Zabaleta et al. 2016). In this strain, the overexpression of native *yciAEc* together with nitrogen depletion and a glucose feed yielded 3HB product titers of up to 14.3 g/L (Guevara-Martínez et al. 2019). A second example for the biotechnological application of YciA is the production of dicarboxylic acids with *Methylorubrum extorquens.* Here, YciA originating from *E. coli* (YciAEc) or *Haemophilus influenzae* (YciAHI) was used to produce 2-methylsuccinic acid and mesaconic acid from CoA-ester intermediates of the ethylmalonyl-CoA-pathway (EMCP) (Sonntag et al. 2014; Pöschel et al. 2022). The authors described the production of a combined titer of 130 mg/L of 2-methylsuccinic acid and mesaconic acid in shake flask cultures of *M.* *extorquens* expressing *yciAEc* (Sonntag et al. 2014), which could be improved to 247 mg/L in a *yciAHI* expressing strain by minimizing product reuptake by deletion of transporter gene *dctA2* (Pöschel et al. 2022). These applications show the potential of thioesterase YciA in biotechnological processes. The broad range thioesterase YciAHI shows activity in a “physiologically relevant” range (*k*_cat_/*K*_m_ > 10^4^ M^−1^ s^−1^) for a wide range of acyl-CoA thioesters (Zhuang et al. 2008) and is therefore a promising catalyst for release of various CoA-bound acids.

The crystal structure of YciAHI was elucidated and assigned the enzyme to the hot dog fold thioesterase superfamily (Willis et al. 2008). The characteristic hot dog fold motif consists of a five-stranded, antiparallel ß-fold wrapped around an elongated α-helix (Dillon and Bateman 2004; Zhuang et al. 2008). This fold diverged from an ancient archetype to fulfill a plethora of functions in the cell. Dillon and Bateman identified 18 different domain architectures for hot dog fold proteins and defined 17 subfamilies, eight of them being thioesterases (Dillon and Bateman 2004). The 3D architecture of this motif itself is highly conserved (Pidugu et al. 2009). Nevertheless, the well-studied 4-hydroxybenzoyl-CoA thioesterases from *Arthrobacter* sp. and *Pseudomonas* sp. give an example of how diverse these enzymes can be composed. The two thioesterases differ in structure of the CoA binding site, quaternary association, and the position of the catalytic residues, while showing a similar hot dog fold topology and acting on the same native substrate (Schmitz et al. 1992; Dunaway-Mariano and Babbitt 1994; Zhuang et al. 2003). In addition to the diversity in tertiary monomer structures, the superfamily of hot dog fold thioesterases includes a multitude of oligomeric structures as dimers, tetramers, and hexamers with divergent inter-monomer orientation (Pidugu et al. 2009).

The hexameric structure of YciAHI is composed of a trimer of dimers, which each harbors two symmetrical equivalent binding sites for CoA-bound substrates at the dimer interface (Willis et al. 2008). The key catalytic amino acid D44 is located in a depression that accommodates the acyl chain of the ligand. This depression is formed by residues K52, 58–61, and 125–131 of monomer A and residues 29–35 of monomer B (Zhuang et al. 2008). The accessibility of the acyl-binding site for different substrates most probably determines the kinetic parameters for the individual substrates. Modification of this part of YciAHI might affect the affinity towards certain substrates.

In this study, we engineered the substrate binding site of YciAHI aiming for improved production of dicarboxylic acids from EMCP-CoA-ester intermediates*.*

## Material and methods

### Growth conditions of bacterial cultures

*E. coli* cultures were grown in LB medium (Bertani 1951) at 37 °C, and kanamycin sulfate was added at 30 μg/mL, if required. For cultivation of *M. extorquens* AM1 (DSM 1338, Peel and Quayle 1961), methanol minimal medium was prepared as described before (Peyraud et al. 2009), containing a CoCl_2_ concentration of 12.6 µM (Kiefer et al. 2009; Sonntag et al. 2014) and 123 mM methanol as carbon source. If required, 50 μg/mL of kanamycin sulfate was added to the medium. For preparation of solid medium, 15 g/L agar-agar was added to the LB or methanol minimal medium. For cultivation of *M. extorquens* AM1 in liquid medium, precultures of 5 mL were grown in test tubes for 48 h at 30 °C and 180 rpm on a rotary shaker. For the preparation of cell extracts, main cultures of 60 or 400 mL were inoculated to an OD_600_ of 0.1 in shake flasks and grown at 30 °C and 180 rpm on a rotary shaker. For the testing of *yciAHI* expression plasmid libraries, main cultures of 1 mL were inoculated to an OD_600_ of 0.1 and grown in deep well plates in a Microtron rotary shaker (Infors, Bottmingen, Switzerland) at 900 rpm. From these cultures, 100 µL were sampled in a microtiter plate and were measured for scattered light signal on a SPARK® multimode microplate reader from TECAN (Männedorf, Switzerland).

### Chemicals

If not stated differently, chemicals were purchased from Carl Roth (Karlsruhe, Germany), Merck (Darmstadt, Germany), or VWR International (Darmstadt, Germany). Substrate (2*S*)-methylsuccinyl-CoA was synthesized by GenoSynth (Berlin, Germany). DL-β-hydroxybutyryl-CoA lithium salt was bought from abcr (Karlsruhe, Germany); acetyl-CoA sodium salt was bought from Cayman Chemical (Michigan, USA).

### Molecular docking of YciAHI and CoA ligands and design of yciAHI expression plasmid libraries

A docking simulation of YciAHI (*H. influenzae*) crystal structure (PDB entry 1YLI) and ligand was done in UCSF Chimera (Pettersen et al. 2004) using AutoDock Vina (Trott and Olson 2010). Prior to docking, the ligand (CoA) and all excess molecules such as solvents were deleted from the protein crystal structure. Hydrogens and charges were assigned to the new ligand (Gasteiger method) and the protein (AMBER force fields). Out of the resulting dockings, the four conformations with highest score (based on the hydrogen bonds formed) were selected. We then identified amino acids that might influence the substrate binding based on the following information: (1) the four selected docking models, (2) the crystal structure of *Thermus thermophilus* acyl-CoA thioesterase PaaI with bound hexanoyl-CoA (PDB entry 1WN3, Kunishima et al. 2005), (3) the YciAHI crystal structure bound to CoA (PDB entry 1YLI), and (4) the predicted location of uncleaved substrates of YciAHI from literature (Willis et al. 2008). From the set of selected amino acids, the hydrophobic ones were substituted for other hydrophobic or neutral amino acids. The corresponding gene variant sequences were synthesized and subcloned in pCM160_RBS_*yciAHI* by BioCat (Heidelberg, Germany). A saturation mutagenesis for position 35 was performed with the Q5® Site-Directed Mutagenesis Kit according to the manufacturer’s protocol (NEB, Frankfurt, Germany). In this second pCM160_RBS_*YciAHI* library, the DNA triplet coding for phenylalanine at position 35 was exchanged with triplets encoding alternative amino acids (A, D, E, G, H, I, K, M, N, P, Q, S, T, Y). Oligonucleotides used for introducing mutations are listed in Table 1. All constructs were confirmed by Sanger sequencing.

**Table 1:** Strains, plasmids, and oligonucleotides used in this work. Bold and underlined letters indicated introduced mutations in pCM160_RBS_*yciAHI*. Oligonucleotides were purchased from Merck (Darmstadt, Germany)

Name	Description	Reference
Bacterial strains		
*E. coli* DH5α	F^–^ φ80*lac*ZΔM15 Δ(*lac*ZYA-*arg*F)U169 *rec*A1 *end*A1 *hsd*R17(r_K_^–^, m_K_^+^) *pho*A *sup*E44 λ^–^*thi*-1 *gyr*A96 *rel*A1	Hanahan 1985; Grant et al. 1990
*E. coli* BL21 (DE3)	*fhuA2 [lon] ompT gal (λ DE3) [dcm] ∆hsdS* *λ DE3* = *λ sBamHIo ∆EcoRI-B int::(lacI::PlacUV5::T7 gene1) i21 ∆nin5*	NEB (Frankfurt, Germany)
*E. coli* AF1000	*MC4100, relA* +	Sandén et al. 2003
*M. extorquens* AM1 (DSM 1338)	Cm^R^, Gram-negative, facultative methylotrophic, obligate aerobic, α-proteobacterium	Peel and Quayle 1961
Plasmids		
pCM160	Kan^R^, p_mxaF_, oriT, pBR322ori, *M. extorquens* expression vector	Marx and Lidstrom 2001
pCM160_RBS_*yciA*HI	pCM160 containing codon-optimized for *M.* *extorquens* thioesterase gene *yciA* from *Haemophilus influenzae*, optimized RBS	Pöschel et al. 2022
pCM160_RBS_*yciAHI*_I34A	pCM160_RBS_*yciAHI* for production of YciAHI variants, modified at positions I34, F35, A48, A51, V59, or V60	This work
pCM160_RBS_*yciAHI*_I34F		
pCM160_RBS_*yciAHI*_F35W		
pCM160_RBS_*yciAHI*_F35L		
pCM160_RBS_*yciAHI*_F35V		
pCM160_RBS_*yciAHI*_A48G		
pCM160_RBS_*yciAHI*_A48V		
pCM160_RBS_*yciAHI*_A48L		
pCM160_RBS_*yciAHI*_A51G		
pCM160_RBS_*yciAHI*_A51V		
pCM160_RBS_*yciAHI*_A51L		
pCM160_RBS_*yciAHI*_V59G		
pCM160_RBS_*yciAHI*_V59L		
pCM160_RBS_*yciAHI*_V59I		
pCM160_RBS_*yciAHI*_V60L		
pCM160_RBS_*yciAHI*_V60I		
pCM160_RBS_*yciAHI*_F35A		
pCM160_RBS_*yciAHI*_F35D		
pCM160_RBS_*yciAHI*_F35E		
pCM160_RBS_*yciAHI*_F35G		
pCM160_RBS_*yciAHI*_F35H		
pCM160_RBS_*yciAHI*_F35I		
pCM160_RBS_*yciAHI*_F35K		
pCM160_RBS_*yciAHI*_F35M		
pCM160_RBS_*yciAHI*_F35N		
pCM160_RBS_*yciAHI*_F35P		
pCM160_RBS_*yciAHI*_F35Q		
pCM160_RBS_*yciAHI*_F35S		
pCM160_RBS_*yciAHI*_F35T		
pCM160_RBS_*yciAHI*_F35Y		
pCM160_RBS_*yciAHI* _FLAG	Vectors for production of C-terminal FLAG-tagged YciAHI variants	This work
pCM160_RBS_*yciAHI*_F35L_FLAG		
pET28a_his6_*yciAHI*	Vectors for production of N-terminal His-tagged YciAHI variants	This work
pET28a_his6_*yciAHI*_F35L		
pJBGT3Rx	*t3* and *rx* from *H.* *boliviensis* under p_lacUV5_ and *lacI* control (p15A/Cm^R^)	Jarmander et al. 2015
pBAD_*zwf*_*yciAHI*	*yciAHI* and *zwf* from AF1000 under control of p_araBAD_ (pBR22/Kan^R^)	This work
pBAD_*zwf*_*yciAHI*_F35L	*yciAHI*_F35L and *zwf* from AF1000 under control of p_araBAD_ (pBR22/Kan^R^)	This work
Oligonucleotides		
LP01_35A_fw	CGGCGACATCGCCGGCGGCTGGA	This work
LP02_35D_fw	CGGCGACATCGACGGCGGCTGGA	
LP03_35E_fw	CGGCGACATCGAGGGCGGCTGGATC	
LP04_35G_fw	CGGCGACATCGGCGGCGGCTGGA	
LP05_35H_fw	CGGCGACATCCACGGCGGCTGGA	
LP06_35K_fw	CGGCGACATCAAGGGCGGCTGGATC	
LP07_35M_fw	CGGCGACATCATGGGCGGCTGGA	
LP08_35N_fw	CGGCGACATCAACGGCGGCTGGA	
LP09_35P_fw	CGGCGACATCCCGGGCGGCTGGATC	
LP10_35Q_fw	CGGCGACATCCAGGGCGGCTGGATC	
LP11_35S_fw	CGGCGACATCTCGGGCGGCTGGA	
LP12_35T_fw	CGGCGACATCACCGGCGGCTGGA	
LP13_35W_fw	CGGCGACATCTGGGGCGGCTGGA	
LP14_35_rev	TTGGCGTTGGTGTCCGAC	
LP15_35I_fw	CGGCGACATCATCGGCGGCTGGA	
LP16_35L_fw	CGGCGACATCCTCGGCGGCTGGA	
LP17_35V_fw	CGGCGACATCGTCGGCGGCTGGA	
LP18_35Y_fw	CGGCGACATCTACGGCGGCTGGA	
LP19_35ILVY_rev	TTGGCGTTGGTGTCCGACGG	

### Plasmid construction

Plasmid DNA was amplified in *E. coli* DH5α and purified with GeneJET Plasmid Miniprep Kit from Thermo Scientific (Waltham, USA). Transformation of *M. extorquens* AM1 was done by electroporation as described before (Toyama et al. 1998). Plasmids for C-terminal FLAG-tagged *yciAHI* expression were constructed by subcloning synthesized *yciAHI-*FLAG inserts (Table 2, Biocat, Heidelberg, Germany) with restriction enzymes SphI and NcoI. For YciaHI protein purification via N-terminal His6-tag, plasmids based on pET28a were constructed by subcloning synthesized his6-*yciAHI* inserts (Table 2, Biocat, Heidelberg, Germany) with restriction enzymes NdeI and EcoRI.

**Table 2:** Synthetic sequences used for plasmid construction. For pCM160 constructs, the codon optimized sequence of *yciaHI* (Pöschel et al. 2022) for *M. extorquens* was used. FLAG tag sequences are written in bold. Restriction sites for subcloning are indicated by underlined letters (*Sph*I and *Nco*I for pCM160 constructs, *Nde*I and *Eco*RI for pET28a constructs)

Name of target construct	Sequence
pCM160_RBS_*yciAHI_*FLAG (C-terminal tagged)	GCATGCCAACAAGTATCTAAAAGATTAAAGGAGGAATAACAATGTCGGCCAACTTCACCGACAAGAACGGCCGCCAGTCGAAGGGCGTCCTCCTCCTCCGCACCCTCGCCATGCCGTCGGACACCAACGCCAACGGCGACATCTTCGGCGGCTGGATCATGTCGCAGATGGACATGGGCGGCGCCATCCTCGCCAAGGAGATCGCCCACGGCCGCGTCGTCACCGTCGCCGTCGAGTCGATGAACTTCATCAAGCCGATCTCGGTCGGCGACGTCGTCTGCTGCTACGGCCAGTGCCTCAAGGTCGGCCGCTCGTCGATCAAGATCAAGGTCGAGGTCTGGGTCAAGAAGGTCGCCTCGGAGCCGATCGGCGAGCGCTACTGCGTCACCGACGCCGTGTTCACCTTCGTCGCCGTGGACAACAACGGCCGCTCGCGCACCATCCCGCGCGAGAACAACCAGGAGCTGGAGAAGGCCCTCGCCCTCATCTCGGAGCAGCCGCTCGACTACAAGGACGACGACGACAAGTGACCATGG
pCM160_RBS_*yciAHI_*F35L_FLAG (C-terminal tagged)	GCATGCCAACAAGTATCTAAAAGATTAAAGGAGGAATAACAATGTCGGCCAACTTCACCGACAAGAACGGCCGCCAGTCGAAGGGCGTCCTCCTCCTCCGCACCCTCGCCATGCCGTCGGACACCAACGCCAACGGCGACATCCTCGGCGGCTGGATCATGTCGCAGATGGACATGGGCGGCGCCATCCTCGCCAAGGAGATCGCCCACGGCCGCGTCGTCACCGTCGCCGTCGAGTCGATGAACTTCATCAAGCCGATCTCGGTCGGCGACGTCGTCTGCTGCTACGGCCAGTGCCTCAAGGTCGGCCGCTCGTCGATCAAGATCAAGGTCGAGGTCTGGGTCAAGAAGGTCGCCTCGGAGCCGATCGGCGAGCGCTACTGCGTCACCGACGCCGTGTTCACCTTCGTCGCCGTGGACAACAACGGCCGCTCGCGCACCATCCCGCGCGAGAACAACCAGGAGCTGGAGAAGGCCCTCGCCCTCATCTCGGAGCAGCCGCTCGACTACAAGGACGACGACGACAAGTGACCATGG
pET28a_his6_*yciAHI* (N-terminal tagged)	CATATGTCTGCCAATTTTACTGATAAAAATGGTCGCCAATCAAAAGGAGTTCTTTTACTACGAACTTTGGCGATGCCTTCTGACACCAATGCTAACGGAGATATTTTTGGTGGCTGGATTATGTCTCAAATGGATATGGGCGGCGCGATTTTAGCGAAAGAAATCGCACACGGACGCGTGGTTACTGTCGCCGTTGAAAGTATGAATTTTATCAAACCAATCTCTGTGGGCGATGTGGTTTGTTGCTACGGTCAATGTCTCAAAGTTGGGCGTTCTTCCATTAAAATTAAAGTAGAAGTATGGGTAAAAAAAGTGGCGAGTGAGCCAATTGGCGAACGTTATTGTGTCACCGATGCGGTATTTACTTTTGTTGCAGTTGATAATAATGGTCGCTCTCGCACGATTCCCCGTGAAAATAACCAAGAGTTAGAAAAAGCATTAGCCTTAATTTCAGAACAACCCTTGTAAGAATTC
pET28a_his6_*yciAHI*_F35L (N-terminal tagged)	CATATGTCTGCCAATTTTACTGATAAAAATGGTCGCCAATCAAAAGGAGTTCTTTTACTACGAACTTTGGCGATGCCTTCTGACACCAATGCTAACGGAGATATTTTAGGTGGCTGGATTATGTCTCAAATGGATATGGGCGGCGCGATTTTAGCGAAAGAAATCGCACACGGACGCGTGGTTACTGTCGCCGTTGAAAGTATGAATTTTATCAAACCAATCTCTGTGGGCGATGTGGTTTGTTGCTACGGTCAATGTCTCAAAGTTGGGCGTTCTTCCATTAAAATTAAAGTAGAAGTATGGGTAAAAAAAGTGGCGAGTGAGCCAATTGGCGAACGTTATTGTGTCACCGATGCGGTATTTACTTTTGTTGCAGTTGATAATAATGGTCGCTCTCGCACGATTCCCCGTGAAAATAACCAAGAGTTAGAAAAAGCATTAGCCTTAATTTCAGAACAACCCTTGTAAGAATTC

### Dicarboxylic acid analysis

*M. extorquens* AM1 cultures were centrifuged for 5 min at 16,000 g, and the supernatants were passed through a 0.22-μm PDVF-syringe filter (Carl Roth, Karlsruhe, Germany). Analysis of the filtered supernatants was performed as described before (Pöschel et al. 2022) on a coupled Nexera X2 UHPLC/LCMS-8045 system (LC-MS/MS, Shimadzu, Duisburg, Germany) equipped with a 150 × 4.6-mm Luna Omega 3 µm PS C18 100 Å column (Phenomenex, Aschaffenburg, Germany). The analytes were negatively ionized with an APCI ion source, fragmented, and finally quantified by comparing the results to calibration curves of peak areas of corresponding standards. Crotonic acid (Carl Roth, Karlsruhe, Germany), (2*S*,3*R*)-2-hydroxy-3-methylsuccinic acid (Enamine, Riga, Latvia), ethylmalonic acid, mesaconic acid, 2-methylsuccinic acid, methylmalonic acid, and succinic acid (Merck, Darmstadt, Germany) were used as analytical standards.

### Preparation of *M. extorquens* crude extracts

A culture (10 mL) was harvested by centrifugation (4 °C, 10 min, 4500 g) in a 5810 R Eppendorf centrifuge (Eppendorf, Hamburg, Germany) at late-exponential growth phase (at about 85% of max. OD_600_). Pellets were resuspended in 600 µL of 20 mM K-HEPES buffer (pH 7.5) premixed with cOmplete™ EDTA-free protease inhibitor cocktail (Roche, Basel, Switzerland). Glass beads (1.1 g of 0.25–0.5-mm beads, previously washed with 5 M NaOH, 5 M HCl, and ddH_2_O) were added, and cells were disrupted at 4 °C for 9 min at 30 Hz in a MM2 mixer mill (Retsch, Haan, Germany). The lysate was centrifuged for 20 min at 4 °C and 14,000 g, and the clear supernatant was analyzed for total protein concentration via BCA assay using the Pierce™ BCA Protein Assay Kit (Thermo Fisher Scientific, Dreieich, Germany).

### SDS PAGE

For SDS PAGEs, the clarified cell extracts were mixed with 4 × Laemmli Sample Buffer, boiled for 5 min, and loaded on an Any kD™ Mini-PROTEAN® TGX™ SDS gel (Bio-Rad, Feldkirchen, Germany). The final samples contained 20 µg of total soluble protein. The PageRuler™ Prestained Protein Ladder (Thermo Fisher Scientific, Dreieich, Germany) was used as a size standard. The SDS PAGE was run at 80 V for 20 min and subsequently at 120 V for 40 min. If required, gels were stained with SimplyBlue™ SafeStain (Thermo Fisher Scientific, Dreieich, Germany).

### Semi-quantitative detection of FLAG-tagged proteins

Immunoblotting of unstained SDS gels was done on a PVDF membrane in a Mini Trans-Blot® Electrophoretic Transfer Cell, following the manufacturer’s protocol for high intensity field transfer (Bio-Rad, Feldkirchen, Germany). A transfer buffer containing 25 mM Tris, 192 mM glycine, and 20% v/v methanol at pH 8.05 was used. Immunodetection was done with monoclonal ANTI-FLAG M2-Alkaline Phosphatase Clone M2 (Merck, Darmstadt, Germany) following the manufacturer’s protocol. The detection buffer (pH 9.5) was prepared by dissolving a SIGMAFAST™ BCIP/NBT tablet (Merck, Darmstadt, Germany) in 10 mL deionized water. The washed PVDF membrane was developed in the detection buffer for 50 s. The reaction was stopped by washing the membrane in distilled water.

### Production, purification, and quantification of his-tagged YciAHI

For protein production, 200 mL of *E. coli* BL21(DE3) (NEB, Frankfurt, Germany) cultures harboring pET28a_his6_*yciAHI* or pET28a_his6_*yciAHI*_F35L were inoculated from precultures to an OD_600_ of 0.1 and incubated at 37 °C. His6-*yciAHI* expression was induced by adding 0.4 mM of IPTG when OD_600_ reached 0.6. Production of N-terminal His-tagged YciAHI took place overnight at 18 °C. Cultures were chilled on ice for 5 min before centrifuging the cells for 30 min at 4 °C and 4500 g. Cell pellets were resuspended in 15 mL of equilibration buffer (20 mM K-HEPES (pH 7.5), 0.5 M NaCl, 10 mM imidazole, pH 7.5) containing cOmplete™ EDTA-free protease inhibitor cocktail (Roche, Basel, Switzerland) and 1 mg/mL lysozyme powder (~ 70,000 U/mg, Merck, Darmstadt, Germany). Cells were chilled on ice for 30 min and disrupted with a probe sonicator (4 min total, 0.5-s pulse, 1-s pause, 25% amplitude). The lysate was centrifuged at 4000 g for 25 min at 4 °C. Purification of N-terminal His-tagged proteins from the supernatant was done using HisPur™ Ni-NTA Spin Columns with 3 mL resin bed (Thermo Fisher Scientific, Dreieich, Germany) according to the manufacturer’s protocol with the following buffers. Equilibration buffer: 20 mM K-HEPES (pH 7.5) containing 0.5 M NaCl and 10 mM imidazole, wash buffer 1/2/3: 20 mM K-HEPES (pH 7.5) containing 0.5 M NaCl and 40/50/60 mM imidazole, respectively, elution buffer: 20 mM K-HEPES (pH 7.5) containing 0.5 M NaCl and 250 mM imidazole. Imidazole concentration was reduced in the final samples by exchanging the elution buffer to protein storage buffer (20 mM K-HEPES (pH 7.5) containing 0.15 M NaCl) with Amicon Ultra-15, 3 MWCO (Merck, Darmstadt, Germany). Protein concentration was measured with Pierce™ BCA assay (Thermo Fisher Scientific, Dreieich, Germany).

### DTNB thioesterase assay

The activity of N-terminal His-tagged YciAHI and YciAHI_F35L towards different CoA-thioesters was determined with a modified DTNB spectrophotometric assay (Ellman 1959; Zhuang et al. 2008). During establishment of the assay, the assay protein concentration was determined, and protein concentration dependence was ensured. The final assay mixture containing 1 mM DTNB, 150 mM NaCl, 100 mM K-HEPES, 0.75 µM purified thioesterase, and 0–150 µM CoA-thioester at pH 7.5 was set up as follows in a total reaction volume of 250 µL. First, 100 µL of assay buffer (108.9 mM K-HEPES, 337.5 mM NaCl, pH 7.5) was premixed with 25 µL DTNB stock solution (108.9 mM K-HEPES, 10 mM DTNB, pH 7.5) in a disposable micro cuvette. Subsequently, 100 µL of CoA-ester solution in 108.9 mM K-HEPES (pH 7.5) was added to the cuvette, and the mixture was incubated at 25 °C for 1 min. For starting the reaction, 25 µL of protein solution (containing 20 mM K-HEPES, 150 mM NaCl, 7.5 µM purified protein, pH 7.5) was added to the assay. The formation of 5-thio-2-nitrobenzoate was monitored by measuring the absorbance at 412 nm. Crude cell extracts were assayed accordingly, using a DTNB stock solution containing 4 mM of DTNB and no NaCl. For starting the reaction, 25 µL of clarified lysate containing 20 µg of protein in 20 mM K-HEPES buffer (pH 7.5) was used.

### Production of (R)-3-hydroxybutyrate by overexpression of *yciAHI* variants in *E. coli*

Shake flask seed cultures of *E. coli* AF1000 containing pJBGT3Rx and additionally either pBADzwf_*yciAHI* or pBADzwf_*yciAHI*_F35L were inoculated from a − 80 °C glycerol stock and cultivated at 37 °C and 180 rpm. The cultivation medium used was a minimal salt medium consisting of 5 g/L (NH_4_)_2_SO_4_, 1.6 g/L KH_2_PO_4_, 6.6 g/L Na_2_HPO_4_·2H_2_O, and 0.5 g/L (NH_4_)_2_-H-citrate and separately sterilized components as 5 g/L glucose, 1 mL/L of 1 M MgSO_4_·7H_2_O solution, 50 mg/L kanamycin (AppliChem Panreac, Darmstadt, Germany), 25 mg/L chloramphenicol (Sigma-Aldrich), and 1 mL/L trace element stock solution (Guevara-Martínez et al. 2019). The trace element stock solution consisted of 0.5 g/L CaCl_2_·2H_2_O, 16.7 g/L FeCl_3_·6H_2_O, 0.18 g/L ZnSO_4_·7H_2_O, 0.16 g/L CuSO_4_·5H_2_O, 0.15 g/L MnSO_4_·4H_2_O, 0.18 g/L CoCl_2_·6H_2_O, and 20.1 g/L Na_2_-EDTA (Guevara-Martínez et al. 2019). Overnight cultures were harvested (4030 g, 10 min), resuspended, and subsequently used to inoculate 800 mL of fresh medium in 1-L stirred tank bioreactors to an OD of 0.1 as described before (Guevara-Martínez et al. 2019). The cultivation medium was the same medium used for the shake flasks seed cultures, except it contained 1.28 g/L (NH_4_)_2_SO_4_, 0.7 g/L Na_3_C_6_H_5_O_7_·2H_2_O, and 12 g/L glucose and no (NH_4_)_2_-H-citrate. For the induction of recombinant expression, 200 μM IPTG and 0.002%(w/w) L-arabinose were used. Samples taken during cultivation were filtered through a 0.2-µm syringe filter, and corresponding supernatants were analyzed. Quantification of glucose, 3HB, and acetic acid was done by ion exchange HPLC on an HPX-87H organic acid column (Bio-Rad, Hercules, Canada) using the refractive index (RI) as described before (Guevara-Martínez et al. 2019). Ammonium concentrations were determined using the Ammonia assay Kit K-AMIAR (Megazyme, Leinster, Ireland). The cell dry weight (CDW) value is a product of OD_600_ multiplied by 2.7 (previously determined).

### Alignment of protein sequences

Sequences were acquired from the ThYme database (Cantu et al. 2010; Caswell et al. 2022) and aligned using the COBALT alignment tool (Papadopoulos and Agarwala 2007). Results were presented with the SnapGene software (GSL Biotech LLC, San Diego, Canada).

## Results

### Screening of a semi-rational mutant library of thioesterase YciAHI

**Fig. 1:**
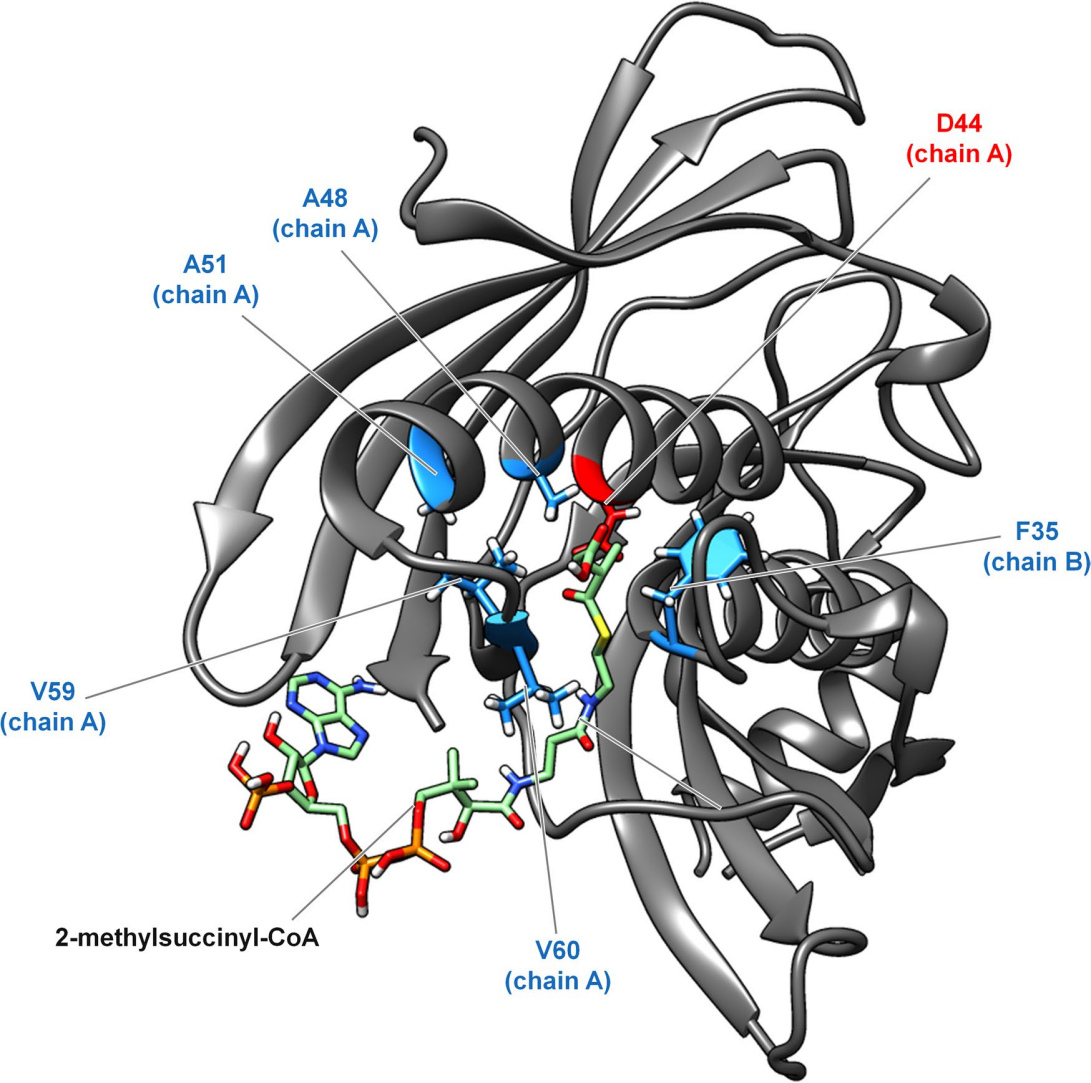
Molecular docking model of (2*S*)-methylsuccinyl-CoA and YciAHI with targeted amino acids. The ligand is docking at the interface of two hot dog folded monomers. The shown protein section consists of parts of monomer A (amino acid 11–125) and monomer B (amino acid 14–151). Catalytic residue D44 is colored in red; amino acids selected for exchange are colored in light blue. The respective chain is indicated in brackets. For better illustration, only one of the two symmetrically equivalent active site channels of the dimer is highlighted

*M. extorquens* AM1 strains can be used to produce a variety of products including the dicarboxylic acids 2-methylsuccinic acid and mesaconic acid (Sonntag et al. 2014, 2015; Pöschel et al. 2022). In these studies, the broad range thioesterase YciAHI is the key enzyme, that hydrolyzes the EMCP intermediates: methylsuccinyl-CoA and mesaconyl-CoA. Aiming at YciAHI variants with the capability to hydrolyze other CoA-esters of the EMCP or with improved productivity, we tried to engineer the substrate-binding region of the enzyme. We identified hydrophobic amino acids that potentially interact with substrates using several docking models of YciAHI and 2-methylsuccinyl-CoA as well as crystal structures and overlays of models from literature (see Material and methods for detailed information). Figure 1 represents an example of a model generated in our docking studies, highlighting the catalytic residue and amino acids selected for exchange. We designed a small semi-rational *yciaHI* expression plasmid library encoding enzyme variants deviating from the wild type YciAHI enzyme at one of the selected positions. The selected hydrophobic amino acids were each exchanged for larger and smaller hydrophobic amino acids to investigate possible steric limitations of the substrate channel of the wild type enzyme.

**Fig. 2:**
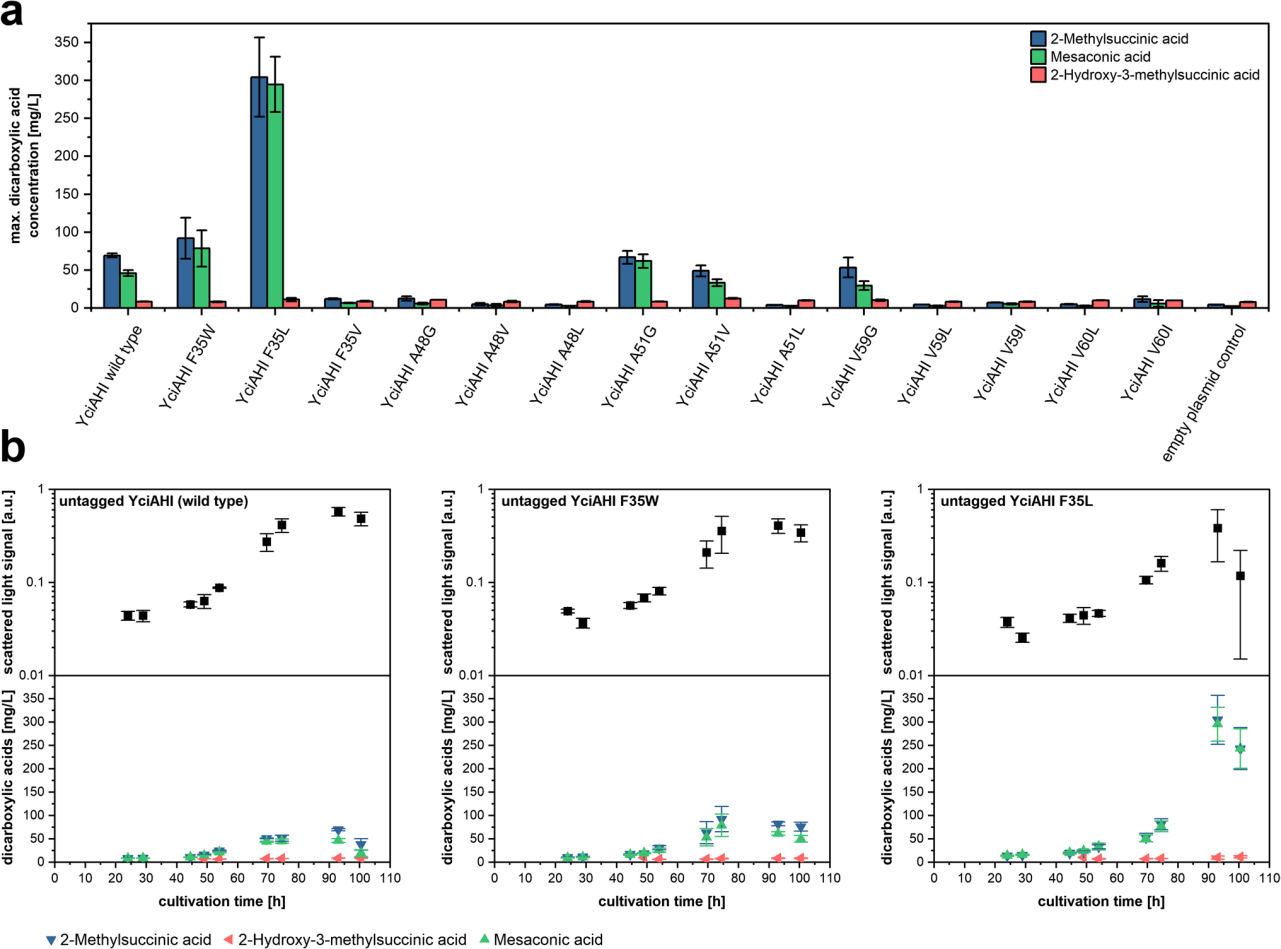
Production of 2-methylsuccinic acid and mesaconic acid with YciAHI variants in *M. extorquens* AM1 in vivo. **a** Maximum product titers of 2-methylsuccinic acid, mesaconic acid, and 2-hydroxy-3-methylsuccinic produced by *M. extorquens* AM1 harboring YciAHI variants. **b** Growth kinetics and time-dependent concentration of mesaconic acid, 2-methylsuccinic acid, and 2-hydroxy-3-methylsuccinic in supernatants of *M.* *extorquens* AM1 harboring YciAHI, variant F35W, or variant F35L (all untagged). Error bars represent standard deviations from three independent replicates. Production kinetics for all strains are shown in Online Resource Fig. S1

We tested the mutant enzymes with the amino acid exchanges in the binding pocket in in vivo production experiments in *M. extorquens* AM1. For the expression of the respective *yciAHI* gene variants, plasmids based on pCM160, a vector for strong constitutive expression, were designed. Plasmids for the production of YciAHI and variants F35W, F35V, F35L, A48G, A48V, A48L, A51G, A51V, A51L, V59G, V59L, V59I, V60L, or V60I, as well as an empty pCM160 plasmid control were introduced in *M. extorquens* AM1, and the release of dicarboxylic acids into the culture medium was analyzed. While the concentration of other EMCP-derived dicarboxylic acid products was below the quantification limit, the titers of 2-methylsuccinic acid, mesaconic acid, and 2-hydroxy-3-methylsuccinic acid could be quantified. The majority of YciAHI modifications lowered the amount of released dicarboxylic acid products in comparison to the wild type enzyme (Fig. 2a). Strains with YciAHI variants A51V and V59G showed similar maximum titers of 2-methylsuccinic acid and mesaconic acid compared to strains harboring unmodified YciAHI, whereas the maximum mesaconic acid titer of strains with YciAHI variant A51G was increased 1.3-fold. The most notable amino acid position was F35. Here, the F35V exchange led to a very low maximal titer of both 2-methylsuccinic acid and mesaconic acid. In contrast, exchanging F35 for W led to an increase in maximum product titer of 1.3-fold for 2-methylsuccinic acid and 1.7-fold for mesaconic acid. The YciAHI variant F35L even exceeded those values by increasing the amount of 2-methylsuccinic acid released 4.4-fold to 304 ± 52 mg/L, and mesaconic acid released 6.4-fold to 295 ± 36 mg/L. The growth kinetics of strains harboring the high level producing YciAHI F35 variants and those harboring the wild type enzyme followed a similar pattern, but F35 strains showed a slightly increased lag phase, a lower growth rate, and a decreased cell density at the end of cultivation (Fig. 2b).

These experiments indicated that the F35 position may be important for substrate conversion rate and/or binding efficiency for YciAHI and its ligands. Unexpectedly, none of the enzyme variants led to a significantly enhanced release of 2-hydroxy-3-methylsuccinic acid or other EMCP-derived carboxylic acids.

### Investigation of YciAHI and YciAHI F35L enzyme levels via FLAG-tag

**Fig. 3:**
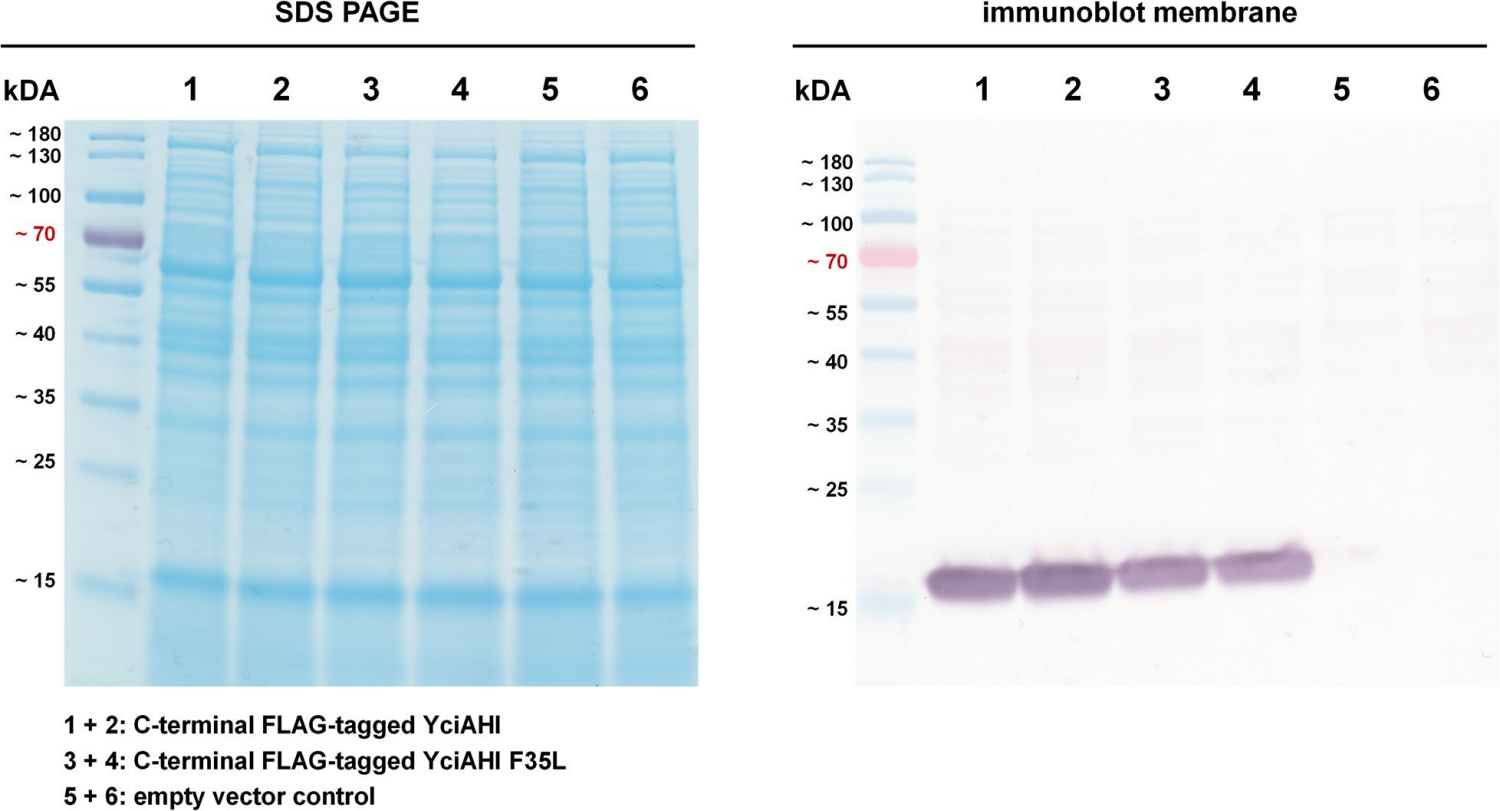
Semi quantitative detection of C-terminal FLAG-tagged YciAHI and C-terminal FLAG-tagged YciAHI F35L in cell extracts of *M. extorquens* AM1. Coomassie stained SDS-PAGE gel and immunoblot membrane of whole protein extracts (20 µg of total protein per lane) of *M. extorquens* AM1 expressing *yciaHI_*FLAG, *yciaHI_*F35L_FLAG or harboring an empty pCM160 vector control. The PVDF membrane was treated with monoclonal ANTI-FLAG M2-Alkaline phosphatase antibody and stained with BCIP/NBT. Duplicate samples were harvested from two individual strains. Growth and dicarboxylic acid production kinetics for all strains are shown in Online Resource Fig. S2

To test whether a higher cellular enzyme concentration is causative for the observed phenotype of strains harboring YciAHI F35L, protein levels were determined on a semi quantitative level. For this purpose, *M.* *extorquens* AM1 cells expressing a FLAG-tag-containing version of the genes *yciAHI* or *yciAHI*_F35L together with an empty vector control were cultivated and harvested in the late exponential growth phase. Extracts were prepared and clarified by centrifugation. Subsequently, the clear supernatant of extracted samples was loaded onto SDS gels. Two SDS-PAGEs were performed simultaneously, generating two identical gels, containing 20 µg of total protein per lane. One of the gels was stained, and the other gel was blotted on a PVDF membrane. On the stained gel, no apparent differences could be identified between the SDS-PAGE patterns of the total protein extracts of the three different strains (Fig. 3). The blotted membrane was treated with monoclonal ANTI-FLAG M2-Alkaline phosphatase antibodies, and the YciAHI thioesterases were detected with a BCIP/NBT staining reaction. The bands of C-terminal FLAG-tagged proteins were clearly visible and corresponded to the predicted mass of 17.8 kDa (molecular weight of untagged enzyme is 16.8 kDa, Zhuang et al. 2008). The bands of C-terminal FLAG-tagged YciAHI F35L were not as strongly visible as the bands of C-terminal FLAG-tagged YciAHI, which demonstrates lower protein levels of the F35L variant enzyme. Consequently, the substantially higher dicarboxylic acid product titers of strains carrying this enzyme variant (Online Resource Fig. S2) did not originate from higher protein concentrations.

### Determination of activity of YciAHI and YciAHI F35L (untagged) in crude cell extracts

**Fig. 4:**
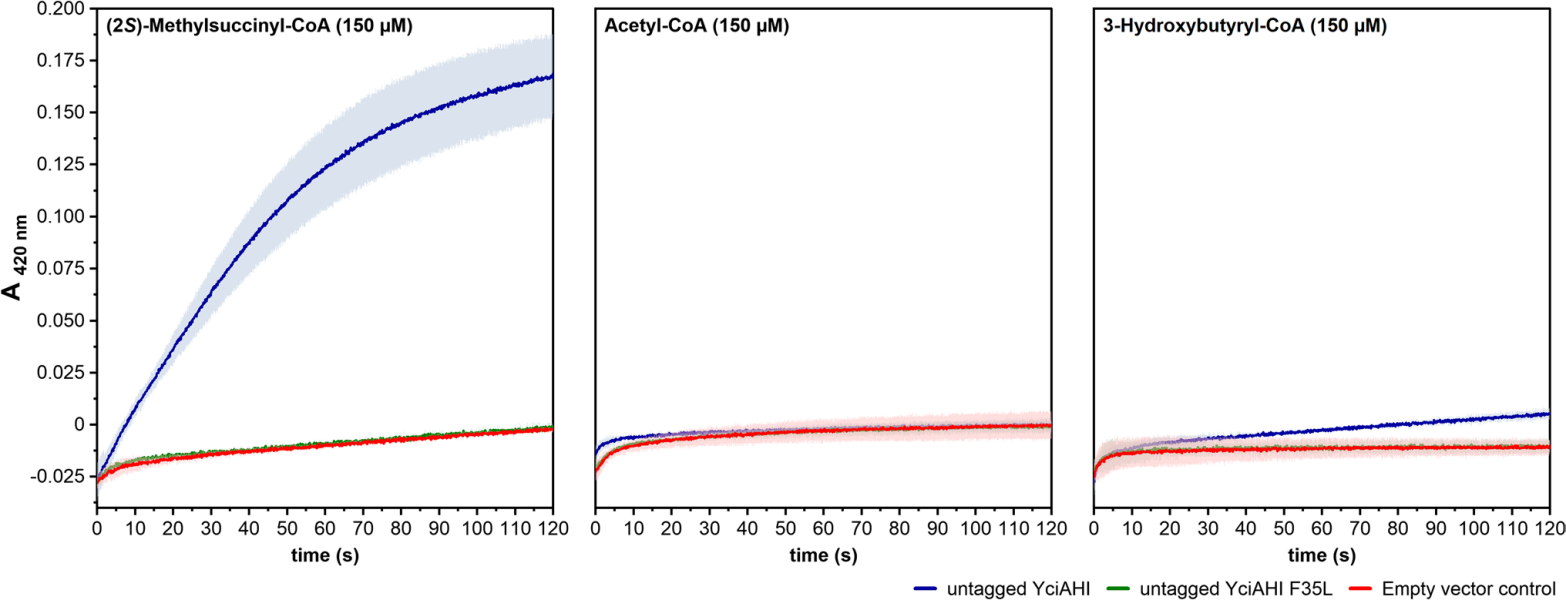
Activity of untagged YciAHI and untagged YciAHI F35L towards EMCP-CoA esters in crude cell extracts. DTNB assays of crude cell extracts of *M. extorquens* overexpressing genes encoding untagged YciAHI or untagged YciAHI F35L or harboring an empty pCM160 vector as a control. As substrate, 150 µM of either (2*S*)-methylsuccinyl-CoA, acetyl-CoA, or 3-hydroxybutyryl-CoA was used. The assay was set up at pH 7.5 in a micro cuvette and contained 0.4 mM DTNB and 20 µg of total protein. The formation of DTNB-derived 5-thio-2-nitrobenzoate was measured at 412 nm. Prior to addition of the lysate, the assay mixture containing all other components was equilibrated to 25 °C. The signal obtained in equilibration phase was set to zero. Lightly colored areas represent standard deviations from three independent replicates

The activity of untagged YciAHI and untagged YciAHI F35L was analyzed in crude cell extracts. *M. extorquens* AM1 was transformed with pCM160_RBS_*yciAHI*, pCM160_RBS_*yciAHI*_F35L, or the empty vector control pCM160, respectively. Cell cultures were harvested in late exponential growth phase, lyzed, and clarified by centrifugation. Thioesterase activity was measured with a photometric DTNB assay. In this assay, DTNB reacts with free CoA to 5-thio-2-nitrobenzoate which is then quantified at 412 nm. Three CoA-ester metabolites were tested, namely (2*S*)-methylsuccinyl-CoA, acetyl-CoA, and 3-hydroxybutyryl-CoA (Fig. 4). With acetyl-CoA, no activity was detected with either cell extract. With (2*S*)-methylsuccinyl-CoA or 3-hydroxybutyryl-CoA, activity was detectable only for cells producing the wild type YciAHI, but not for the cells producing the F35L variant.

### DTNB assay of purified YciAHI and YciAHI F35L (N-terminal His-tagged)

**Fig. 5:**
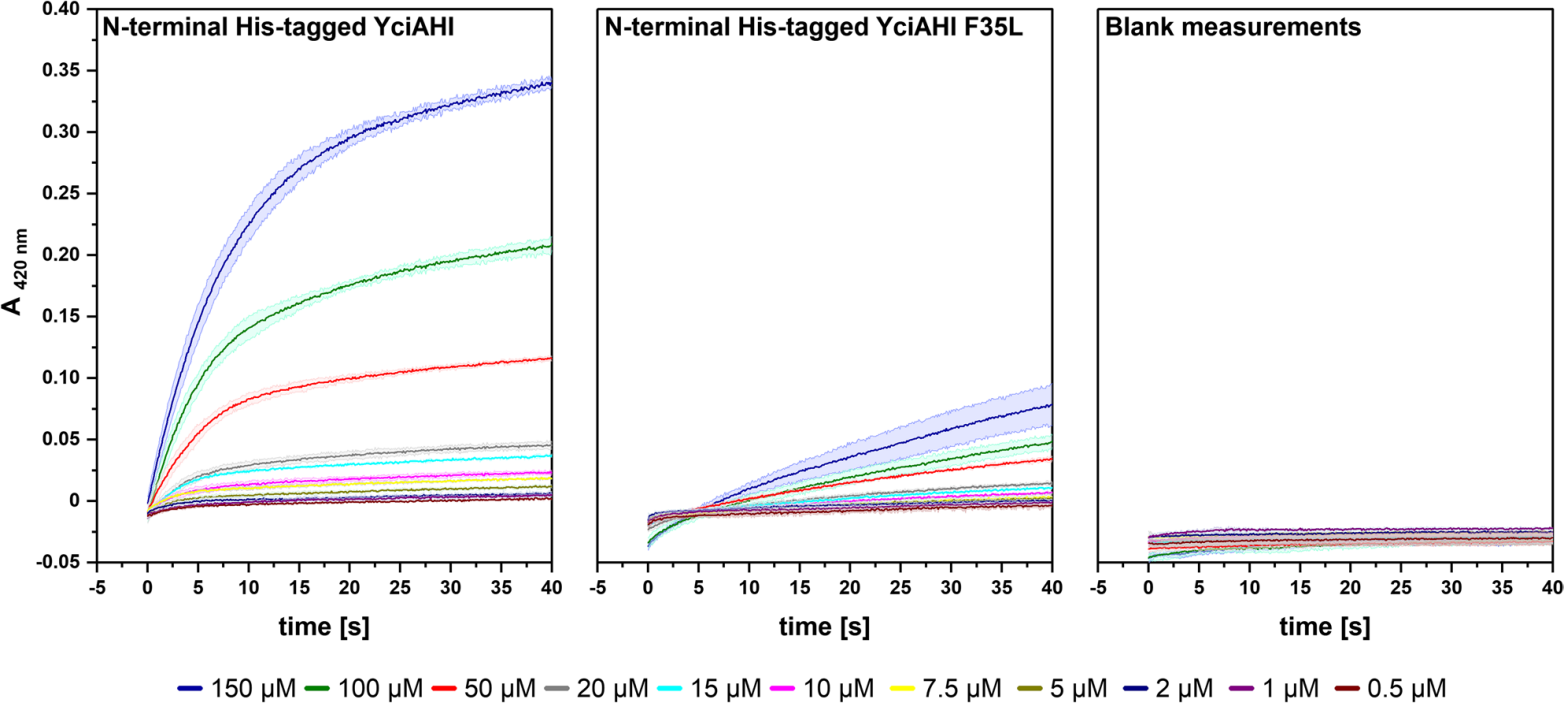
(2*S*)-Methylsuccinyl-CoA conversion tests with purified N-terminal His-tagged YciAHI, N-terminal His-tagged YciAHI F35L thioesterases, and without enzyme addition. Values were determined with a DTNB assay at pH 7.5 and 25 °C using 1–150 µM of (2*S*)-methylsuccinyl-CoA as substrate (color-coded concentrations) and 7.5 µM of purified enzyme. Prior to addition of the protein, the assay mixture containing all other components was equilibrated to 25 °C. For blank measurements, no enzyme was added. The formation of DTNB-derived 5-thio-2-nitrobenzoate was measured at 412 nm. Values were corrected by setting the baseline before protein addition (not shown) to zero. Uncorrected values can be found in Online Resource Fig. S3. All measurements were done in triplicates

Since activity of untagged YciAHI F35L towards the tested CoA-esters was unexpectedly not detectable in crude cell extracts, we additionally tested the purified enzymes. Wild type YciAHI and the F35L variant were produced in *E. coli* and purified via a *his6*-tag. The conversion with different (2*S*)-methylsuccinyl-CoA concentrations was tested in a DTNB assay (Fig. 5).

Substrate concentrations in the physiological sub-µM range for EMCP intermediates (personal communication Patrick Kiefer) and concentrations far beyond the physiological level were tested. The datasets showed a similar behavior to the results acquired with *M.* *extorquens* extracts containing the respective enzymes (Fig. 4). The reaction rates of purified N-terminal His-tagged YciAHI are substantially higher than the values obtained with purified N-terminal His-tagged YciAHI F35L (Fig. 5).

### Application of untagged YciAHI variant F35L for 3-hydroxybutyric acid production with *E. coli*

**Fig. 6:**
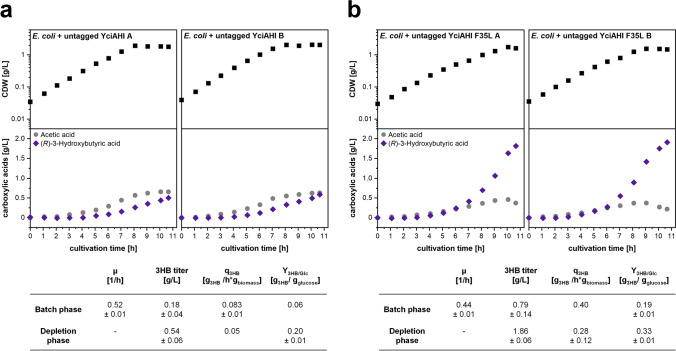
Growth and product formation kinetics during batch cultivations of *E. coli* AF1000 engineered for production of 3HB in vivo. On the expression vectors used, the untagged versions of thioesterases were encoded. All cultivations were performed in duplicates and in parallel. Identical media composition was used in all reactors. Complete nitrogen depletion was detected between 8 and 10 h of cultivation. **a** Duplicates (A and B) of *E. coli* AF1000 containing pJBGT3Rx pBAD_*zwf*_*yciAHI.* **b** Duplicates (A and B) of *E. coli* AF1000 containing pJBGT3Rx pBAD_*zwf*_*yciAHI_F35L*

Although our in vitro enzyme assays failed to verify a higher specific activity of the F35L variant, the improved productivity in the *M.* *extorquens* strain encouraged us to test its utility in another production system. Therefore, we used an *E. coli* strain engineered for 3HB production (Guevara-Martínez et al. 2019) to test YciAHI and the variant YciAHI F35L (both untagged) in vivo in a different environment. We tested *E. coli* AF1000 strains (over-) expressing genes *t3* (encoding a thiolase, WP_007111820), *rx* (encoding a reductase, WP_007111780), *zwf* (encoding a glucose-6-phosphate dehydrogenase, UKY30849), and additionally *yciAHI* or *yciAHI* F35L in an ammonium depleted batch cultivation as already done for the *E. coli yciAEc* gene in previous works (Guevara-Martínez et al. 2019). The cultivation comprised two phases. While in the first phase, biomass and the main product 3HB as well as the by-product acetic acid were formed, in the second, nitrogen-depleted phase (> 8 h of cultivation), the carbon flux towards 3HB production was favored and part of the acetic acid was consumed (Fig. 6). While the strains harboring the YciAHI-wild type enzyme produced up to 0.54 ± 0.06 g/L 3HB (Fig. 6a), the strain harboring the F35L variant produced 1.86 ± 0.06 g/L (Fig. 6b). The 3HB yields (per biomass or glucose) are substantially higher for the F35L variant. These experiments again showed a more than threefold improved productivity caused by the F35L exchange in YciAHI.

### Screening of other YciAHI F35 variants

**Fig. 7:**
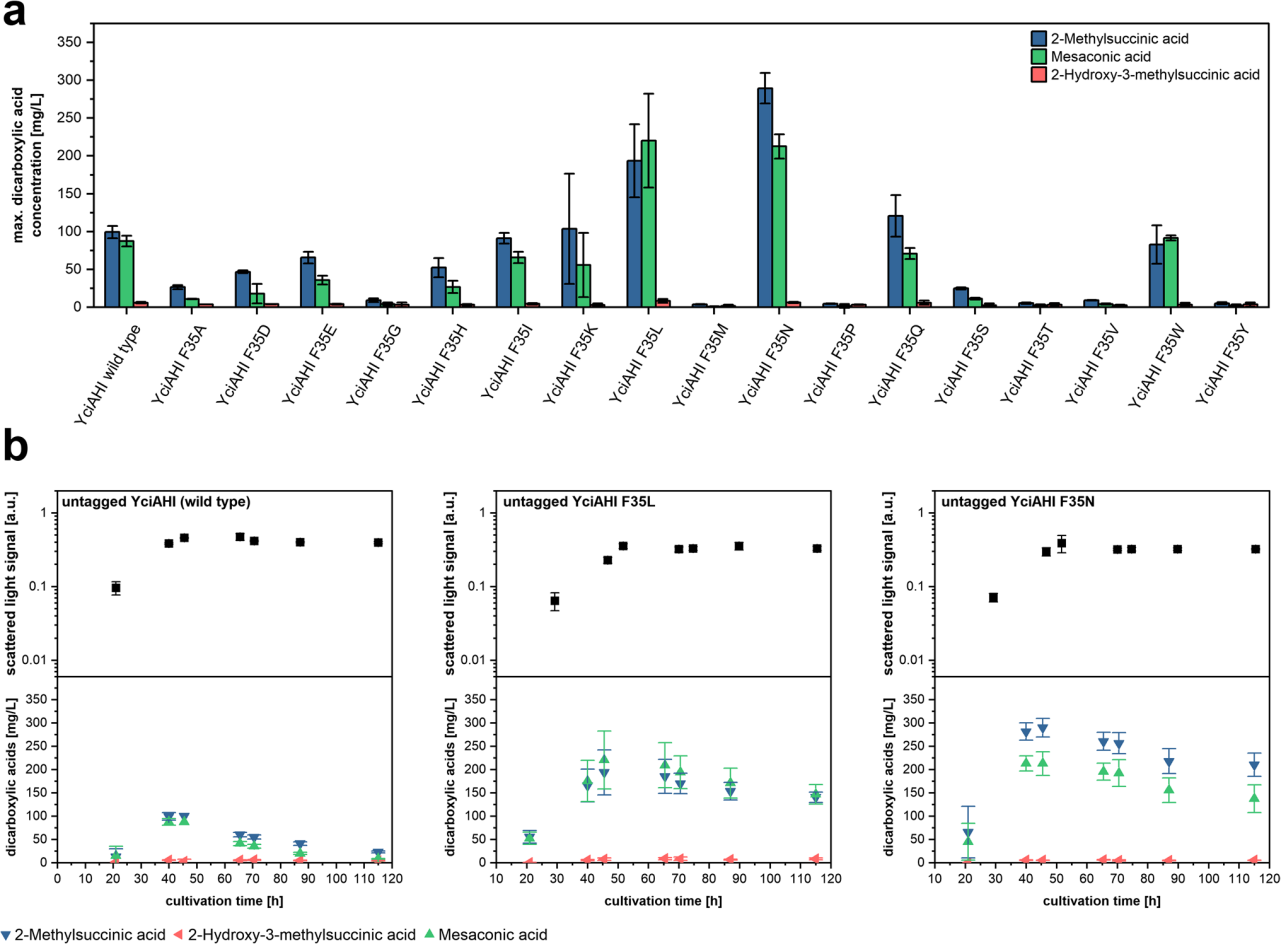
Production of 2-methylsuccinic acid and mesaconic acid with YciAHI F35 variants in *M. extorquens* AM1 in vivo. **a** Maximum product titers of 2-methylsuccinic acid, mesaconic acid, and 2-hydroxy-3-methylsuccinic produced by *M. extorquens* AM1 harboring untagged YciAHI F35 variants. **b** Growth kinetics and time-dependent concentration of mesaconic acid, 2-methylsuccinic acid, and 2-hydroxy-3-methylsuccinic in supernatant of *M.* *extorquens* AM1 harboring YciAHI, variant F35L, or variant F35N (all untagged). Error bars represent standard deviations from three independent replicates. Production kinetics of all strains are shown in Online Resource Fig. S4

As the F35L variant of YciAHI showed enormous productivity improvements in two different biotechnological production systems, we constructed another plasmid library for expression of *yciAHI* by saturation mutagenesis of the F35 position. Except for gene variants *yciAHI*_F35C and *yciAHI*_F35R, we successfully cloned expression constructs for the investigation of all possible YciAHI F35 variants (all untagged). The generated constructs were introduced into *M.* *extorquens* AM1, and the respective strains were cultivated and analyzed for dicarboxylic acid production. Most enzyme variants, namely YciAHI F35A, F35D, F35E, F35G, F35H, F35I, F35K, F35M, F35P, F35S, F35T, and F35V showed reduced or abolished ability to release 2-methylsuccinic acid and mesaconic acid (Fig. 7a). Despite product titers that were slightly higher with unmodified YciaHI and lower with the F35L variant compared to what was observed in Fig. 2, also in this experiment, expression of variant F35L, together with F35N and F35Q, led to substantially increased titers of 2-methylsuccinic acid and mesaconic acid compared to the strain expressing the wild type thioesterase gene. The highest values obtained in the experiments shown in Fig. 7b were obtained with *yciAHI*_F35N resulting in titers of 289 ± 20 mg/L 2-methylsuccinic acid and 213 ± 16 mg/L mesaconic acid after 45.5 h of cultivation.

## Discussion

In two previous studies, several thioesterases for EMCP-derived dicarboxylic acid production were tested in *M.* *extorquens* AM1 by overexpressing the corresponding genes (Sonntag et al. 2014; Pöschel et al. 2022). Out of the seven candidates (TesBec thioesterase B from *E. coli*, TesBext thioesterase B from *M.* *extorquens*, YciAEc ACOT from *E. coli*, YciAHI ACOT from *H.* *influenzae*, ACOT4 succinyl-CoA hydrolase from *Mus* *musculus*, PaaI phenylacetate thioesterase from *Azoarcus evansii*, and Bch 3-hydroxyisobutyryl-CoA hydrolase from *Bacillus cereus*), only strains harboring the YciAEc or YciAHI thioesterase released notable amounts of two EMCP-derived products, namely 2-methylsuccinic acid and mesaconic acid.

The objective of this work was to engineer YciAHI for improved production of EMCP-derived carboxylic acids with *M.* *extorquens.* The YciAEc homolog from *E. coli* was not considered, since up to now, there is no crystal structure available in the publicly accessible databases. We designed YciAHI variants semi-rationally based on docking models of enzyme and ligand and published crystal structures of related thioesterases. For overexpression of the corresponding gene variants, we constructed a small plasmid library and screened for the carboxylic acid production in vivo.

In all *M. extorquens* production experiments, we analyzed the culture supernatants for new EMCP-CoA-ester products such as (2*S*,3*R*)-2-hydroxy-3-methylsuccinic acid, 3HB, ethylmalonic acid, and methylmalonic acid. For none of the YciAHI variants we were able to detect amounts of these products above the quantification limit. Although the concentrations of all CoA intermediates in the EMCP in methanol-grown *M.* *extorquens* AM1 cells are similar (Sonntag et al. 2015), there appears to be no considerable conversion of the potential substrates other than (2*S*)-methylsuccinyl-CoA and mesaconyl-CoA. The semi-rational exchange of hydrophobic amino acids in the binding region of YciAHI carried out in this work does not seem to influence the product spectrum regarding EMCP-derived carboxylic acid products. Predicting substrate selectivity for potential substrates with high similarity as the short-chain EMCP thioesters based on the 3D enzyme structure is rather difficult. Therefore, for accessing new EMCP-derived carboxylic acid products with *M. extorquens* in the future, we suggest a random mutagenesis approach for YciAHI (even neglecting the polarity of residues) followed by in vivo testing or the screening of other heterologous ACOTs with short chain specificity.

In our work, we could identify two variants of YciAHI which led to higher release of 2-methylsuccinic acid and mesaconic acid, namely F35N and F35L, of which the latter was characterized more closely. In general, growth of high-performing strains was impaired as carbon is drained from the anaplerotic EMCP. Although the levels and trends of carboxylic acid production were similar, the exact titers varied between our experiments. This phenomenon may be caused by slightly different growth conditions or product reuptake. The latter could be avoided in the future by using recently described acid transporter deletion mutants (Pöschel et al. 2022).

The observed low in vitro activities of YciAHI F35L measured in this work did not match the in vivo results of substantially higher productivity compared to strains harboring the wild type enzyme. This low activity could indicate that the enzyme may be too unstable for in vitro analysis. We can only hypothesize that the F35L mutation may affect the structural integrity of the hexameric or monomeric structure resulting in an unstable or inactive enzyme under in vitro conditions. There is also the possibility that the observed low activity could be the result of stronger feedback inhibition of the F35L enzyme. Bound CoA or accumulating carboxylic acid products may reduce the activity in the in vitro assays. Unbound CoA in the assay solution should not play a role in this context since it reacts rapidly with DTNB to 5-thio-2-nitrobenzoate.

Although no enhanced productivity for YciAHI F35L could be proven in vitro, we could measure a substantially increased product release in in vivo experiments of 2-methylsuccinic acid and mesaconic acid with *M.* *extorquens* AM1 and of 3HB with *E. coli* production strains. We suspect this to be the result of a change of the conformation of the enzymes binding pocket.

To find possible explanations for the enhanced productivity of YciAHI variants F35L and F35N in vivo, we will discuss the crystal structure of YciAHI and other, closely related thioesterases in the following. Based on their similarity in primary and tertiary structures and the position and identity of the catalytic residues, the superfamily of thioesterases was sorted in families TE1-TE35 in the ThYme database (https://thyme.engr.unr.edu/v2.0/; Cantu et al. 2010; Caswell et al. 2022). YciAHI belongs to the TE6 family ACOTs that act on short- to long-chain acyl-CoAs (C4–C18 products). Prokaryotic TE6 enzymes assemble a functional unit from two identical monomers (topology β1-α1-β2-β3-β4-β5), while eukaryotic TE6 enzymes contain a fused domain of double hotdogs (topology β1-α1-β2-β3-β4-β5-α2-*β6-α3-β7-β8-β9-β10-α4*) as functional unit (Swarbrick et al. 2020). TE6 thioesterases typically co-crystallize with a tightly bound CoA molecule at one of the two potential active sites in a dimer or the equivalent monomeric double hot dog (e.g., PDB entries 1YLI, 1Y7U, 3B7K, 1VPM, Willis et al. 2008; Swarbrick et al. 2014). For YciAHI, the role of CoA as a strong feedback inhibitor was demonstrated by determination of the inhibition constant and attributed to the necessity of regulating the cytoplasmic acyl-CoA hydrolyzing enzyme in the living cell (Zhuang et al. 2008). To date, no structure with an uncleaved substrate is publicly available, so the CoA-bound model (PDB entry 1YLI) is discussed in the following.

YciAHI harbors a hydrophobic pocket in which the thiol group of CoA is tightly anchored. This pocket is not spacious enough to accommodate an acyl-group of a CoA-bound substrate (Willis et al. 2008). Therefore, the thiol group of the thioester substrate must be flipped such that the acyl group is located in the depression leading towards the catalytic amino acid. F35 is located on the edge of the CoA-binding pocket and the depression. From our experiments, we cannot conclude if the higher product release of F35L and F35N variants is caused by changes in the catalytic depression or the hydrophobic CoA pocket.

**Fig. 8:**
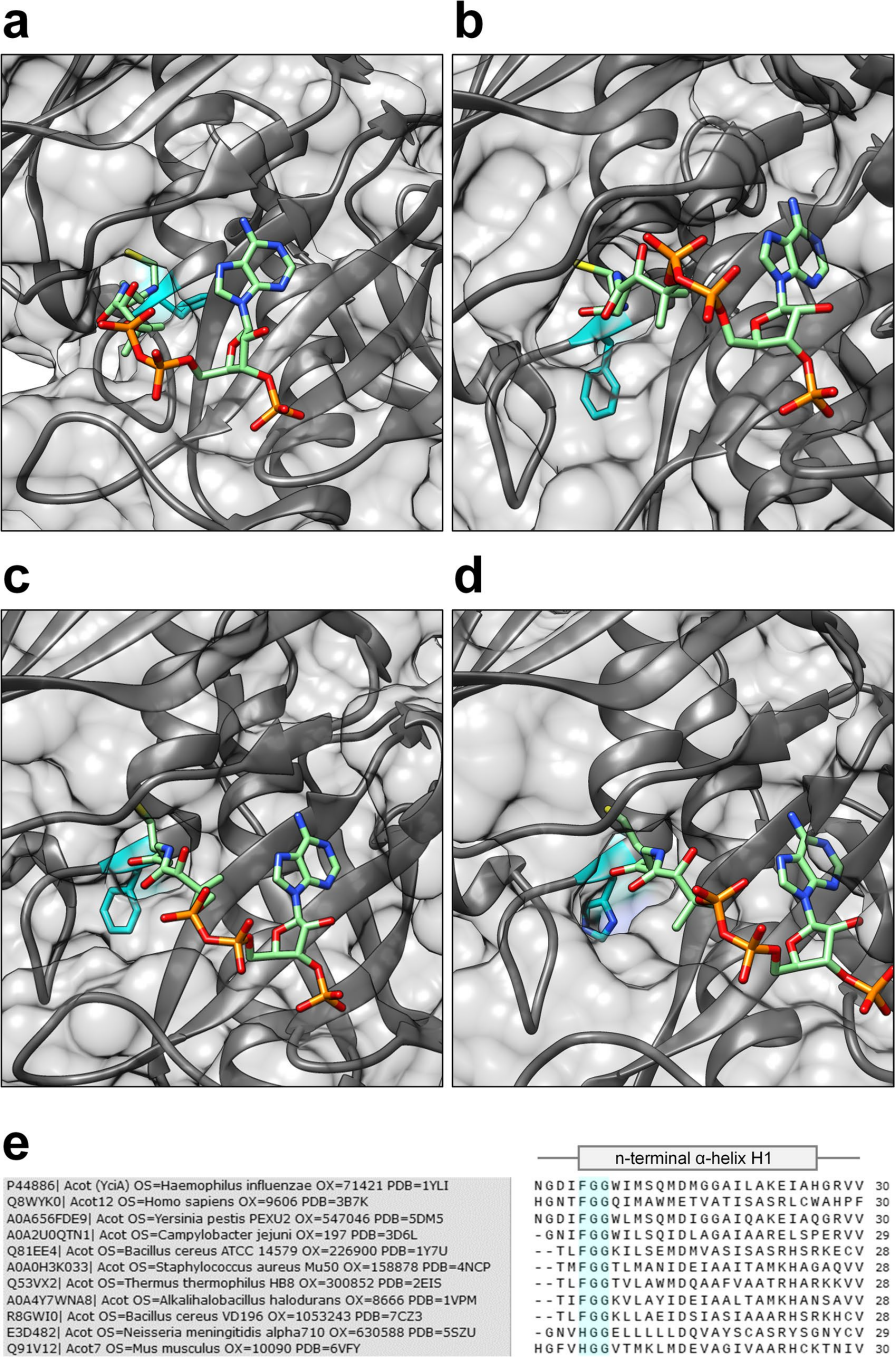
Comparison of CoA binding site of representative TE6 thioesterases YciAHI, ACOT12 (Hs), ACOT (Ah), and ACOT7 (Mm). **a** Crystal structure of YciAHI (*H.* *influenzae*, PDB number 1YLI) with bound CoA. Phenylalanine 35 at the N-terminus of helix α1 is highlighted in blue. **b** Crystal structure of ACOT12 (*H. sapiens*, PDB number 3B7K) with bound CoA. Phenylalanine 201 at the N-terminus of helix α1 is highlighted in blue. **c** Crystal structure of ACOT from *Alkalihalobacillus halodurans* (PDB number 1VPM) with bound CoA. Phenylalanine 30 at the N-terminus of helix α1 is highlighted in blue. **d** Crystal structure of ACOT7 N-domain (*M.* *musculus*, PDB number 2V1O) with bound CoA. Histidine 30 at the N-terminus of helix α1 is highlighted in blue. **e** Sequence alignment of CoA-proximal α-helices of TE6 family members with available crystal structures. Consensus sequence at the N-terminus of the α-helix is highlighted in blue. Sequences, Uniprot identifiers, protein names, organism names (OS), and identifiers (OX), as well as PDB accession numbers were acquired through the ThYme database (https://thyme.engr.unr.edu/v2.0/)

In the crystal structure of YciAHI, F35 is located at the N-terminus of the central α-helix (α1). The phenyl sidechain of F35 is facing away from the binding pocket (Fig. 8a). In all published crystal structures of TE6 ACOTs, the start of α1 (and α3 for eukaryotic enzymes) is highly conserved at sequence (Fig. 8e) and topology level. As example for the latter, crystal structures of eukaryotic ACOT12 (*Homo sapiens*, PDB entry 3B7K) and procaryotic ACOT (*Alkalihalobacillus halodurans*, PDB entry 1VPM) are shown in Fig. 8b and c. As in YciAHI, a phenylalanine residue can be found at the N-terminus of helix α1. In ACOT7 enzymes, instead of a phenylalanine, a histidine residue is positioned at the end of α1 and α3. As an example, the α1 domain of ACOT7 from *Mus* *musculus* (PDB entry 2V1O) is given in Fig. 8d. In all crystal structures available, glycine is marking positions 2 and 3 of the α-helix. An alignment of all TE6 family members from the ThYme database confirms a conserved triplet of F-G-G or H-G-G in the α1/α3 domains (Online Resource Fig. S5). This conservation indicates an important role of the triplet in binding site constitution. Interestingly, the consensus triplet with phenylalanine or equivalent histidine at the start of a α-helix can also be found TE families 4, 7, 8, 11, and 31 (Online Resource Fig. S6-S10). The function of this triplet in other TE families is not part of this study but could be an interesting target for modification of enzymes belonging to these groups.

The comparison of the available TE6 crystal structures indicates that the phenyl or imidazole moiety of the amino acid at the α1 N-terminus does not interact directly with the substrate due to its orientation. Based on a structure overlay of YciAHI and the hexanoyl-CoA ligand from a *H. thermophilus* PaaI model (PDB entry 1WN3), Willis and coworkers speculated that the carbonyl oxygen of the thioester substrate interacts with G36 in analogy to Y24 (*Pseudomonas* enzyme) *or* G65 (*Arthrobacter* enzyme) of 4-hydroxybenzoyl-CoA thioesterases (Willis et al. 2008). Assuming a nucleophilic mode of catalysis, the formation of a hydrogen bond between the backbone NH amide of G36 and the thioester would polarize the C = O group for a nucleophilic attack (Zhuang et al. 2002). Based on our results, we can only hypothesize that the F35 variation may influence the polarization in some way by either taking part itself or bringing G36 in a more favorable position. Another conceivable scenario is that F35 is modulating the size of the CoA binding pocket or the catalytic depression. In either case, we assume that the position of the polypeptide backbone at the N-terminus of helix α1 is crucial. This assumption is also supported by the fact that in our experiments, the physical-chemical side-chain properties of the supplementing amino acid were not the decisive factor for in vivo enzyme activity. F35 could be substituted with nonpolar (F35L, F35I), polar (F35Q, F35W, F35N), acidic (F35E, F35D), or basic (F35K, F35H) amino acids while still remaining active. Instead of chemical properties, rather the length of side chain at the F35 position was critical. For all substitutions tested (except for F35M), it seemed that medium length side chains were favorable for enzyme activity, while short side chains abolished it. Leucine and asparagine might provide the optimal chain length for positioning of the polypeptide backbone.

Although the exact reason for enhanced in vivo characteristics of YciAHI variants F35L and F35N stays unclear, the higher productivity was proven by application in an alternative system. Also in *E. coli* strains engineered for 3HB production, the F35L variant led to substantially higher product titers compared to unmodified YciAHI and even reached the yields and titers of the overexpressed native YciAEc homolog (Guevara-Martínez et al. 2019). With currently available in vitro assay techniques, we would not have been able to identify the variants. Therefore, although it is more laborious, testing new and engineered thioesterases in vivo in the production host can be a valuable strategy*.*

Besides providing more productive YciAHI variants, we contributed new insights into the understanding the structure of TE6 thioesterases. The discussed consensus triplet can be also found in thioesterases from other families such as TesB from TE4 (Swarbrick et al. 2015) or *Arthrobacter* 4-hydroxybenzoyl-CoA from TE11 (Song et al. 2012). A closer examination of this position by investigation of corresponding mutant enzymes might reveal new insights in thioesterase mechanisms in general and might also help to develop improved thioesterase variants for biotechnological applications.

### Supplementary Information

Below is the link to the electronic supplementary material.

Supplementary file1 (PDF 2904 KB)

## Data Availability

All data generated or analyzed during this study are included in this published article and its supplementary information file.
