# Supplementary material for: Engineering of thioesterase YciA from Haemophilus influenzae for production of carboxylic acids
Source: Appl Microbiol Biotechnol. 2023 Aug 12;107(20):6219–36. doi: 10.1007/s00253-023-12691-1 (PMC10560148; doi:10.1007/s00253-023-12691-1)
Supplement: Supplementary file 1 — Supplementary file1 (PDF 2904 KB) [file 253_2023_12691_MOESM1_ESM.pdf]

Online Resource - Supplementary Information

Engineering of thioesterase YciA from *Haemophilus influenzae* for production of carboxylic acids

Laura Pöschel[1,2], Mónica Guevara-Martínez[3], David Hörnström[3], Antonius J.A. van Maris[3], Markus Buchhaupt[1]*

[1] DECHEMA-Forschungsinstitut, Microbial Biotechnology, Theodor-Heuss-Allee 25, 60486 Frankfurt am Main, Germany

[2] Faculty of Biological Sciences, Goethe University Frankfurt, Max-von-Laue-Str. 9, 60438 Frankfurt am Main, Germany

[3] Department of Industrial Biotechnology, School of Engineering Sciences in Chemistry, Biotechnology and Health, KTH Royal Institute of Technology, AlbaNova University Center, SE 10691 Stockholm, Sweden

*Corresponding author,

Phone: +49-69-7564-629, e-mail address: markus.buchhaupt@dechema.de

Fig. S1 Growth kinetics and time-dependent concentration of 2-methylsuccinic acid, 2-hydroxy-3-methylsuccinic acid and mesaconic acid in supernatant of *M. extorquens* AM1 harboring YciAHI variants

Fig. S2 Growth kinetics and time-dependent concentration of 2-methylsuccinic acid and mesaconic acid in supernatant of *M. extorquens* AM1 harboring plasmids encoding YciAHI FLAG, YciAHI F35L FLAG or an empty pCM160 vector

Fig. S3 Uncorrected (2*S*)-methylsuccinyl-CoA conversion tests with purified YciAHI and YciAHI F35L thioesterases

Fig. S4 Growth kinetics and time-dependent concentration of 2-methylsuccinic acid, 2-hydroxy-3-methylsuccinic acid and mesaconic acid in supernatant of *M. extorquens* AM1 harboring YciAHI F35 variants

Fig. S5 Alignment of TE6 family members (α-helix domain)

Fig. S6 Alignment of TE4 family members (α-helix domain)

Fig. S7 Alignment of TE7 family members (presumed α-helix domain)

Fig. S8 Alignment of TE8 family members (α-helix domain)

Fig. S9 Alignment of TE11 family members (α-helix domain)

Fig. S10 Alignment of TE31 family members (α-helix domain)

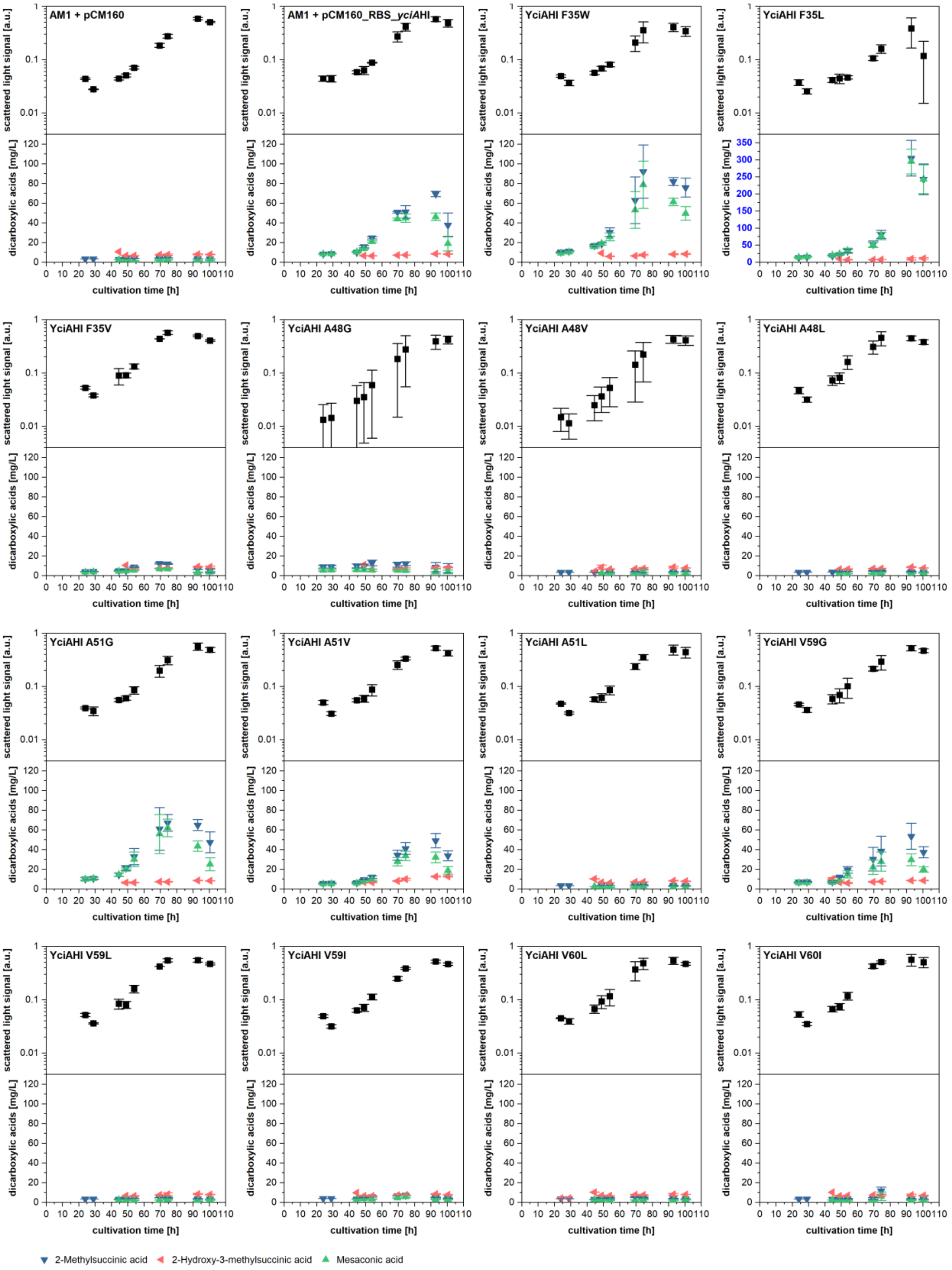

Fig. S1 Growth kinetics and time-dependent concentration of 2-methylsuccinic acid, 2-hydroxy-3-methylsuccinic acid and mesaconic acid in supernatant of *M. extorquens* AM1 harboring YciAHI variants (untagged). EMCP-derived carboxylic acid products with titers insufficient for quantification (< 5 mg/L) are not displayed. Error bars represent standard deviations from three independent replicates

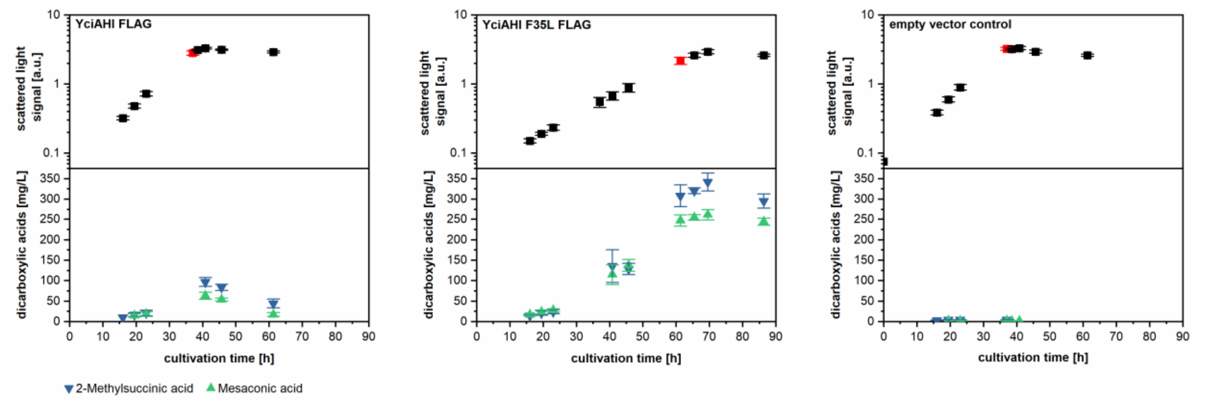

Fig. S2 Growth kinetics and time-dependent concentration of 2-methylsuccinic acid and mesaconic acid in supernatant of *M. extorquens* AM1 harboring plasmids encoding C-terminal FLAG-tagged YciAHI, C-terminal FLAG-tagged YciAHI F35L or an empty pCM160 vector. Red squares indicate the sampling time points for SDS-PAGE samples. Error bars represent standard deviations from three independent replicates

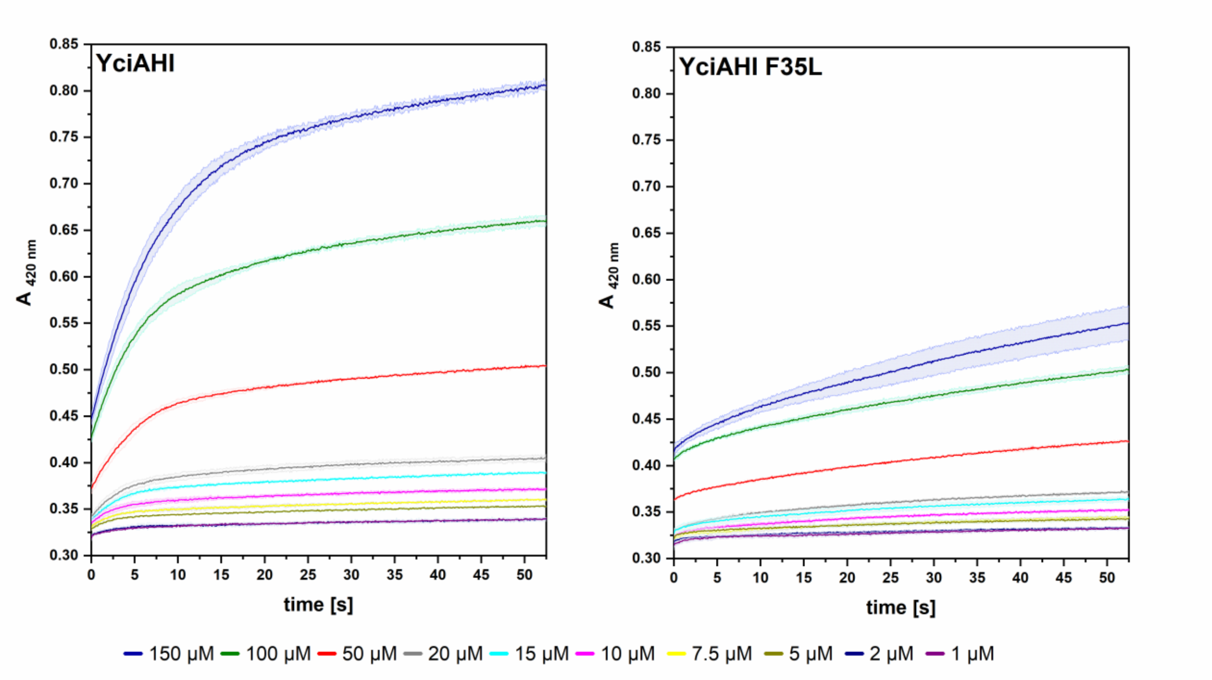

Fig. S3 Uncorrected (2*S*)-methylsuccinyl-CoA conversion tests with purified N-terminal His-tagged YciAHI and N-terminal His-tagged YciAHI F35L thioesterases. Values were determined with a DTNB assay at pH 7.5 and 25°C using 1-150 µM of (2*S*)-methylsuccinyl-CoA as substrate (color coded concentrations) and 7.5 µM of purified enzyme. Prior to addition of the protein, the assay mixture containing all other components was equilibrated to 25 °C. The formation of DTNB-derived 5-thio-2-nitrobenzoate was measured at 412 nm. All measurements were done in triplicates

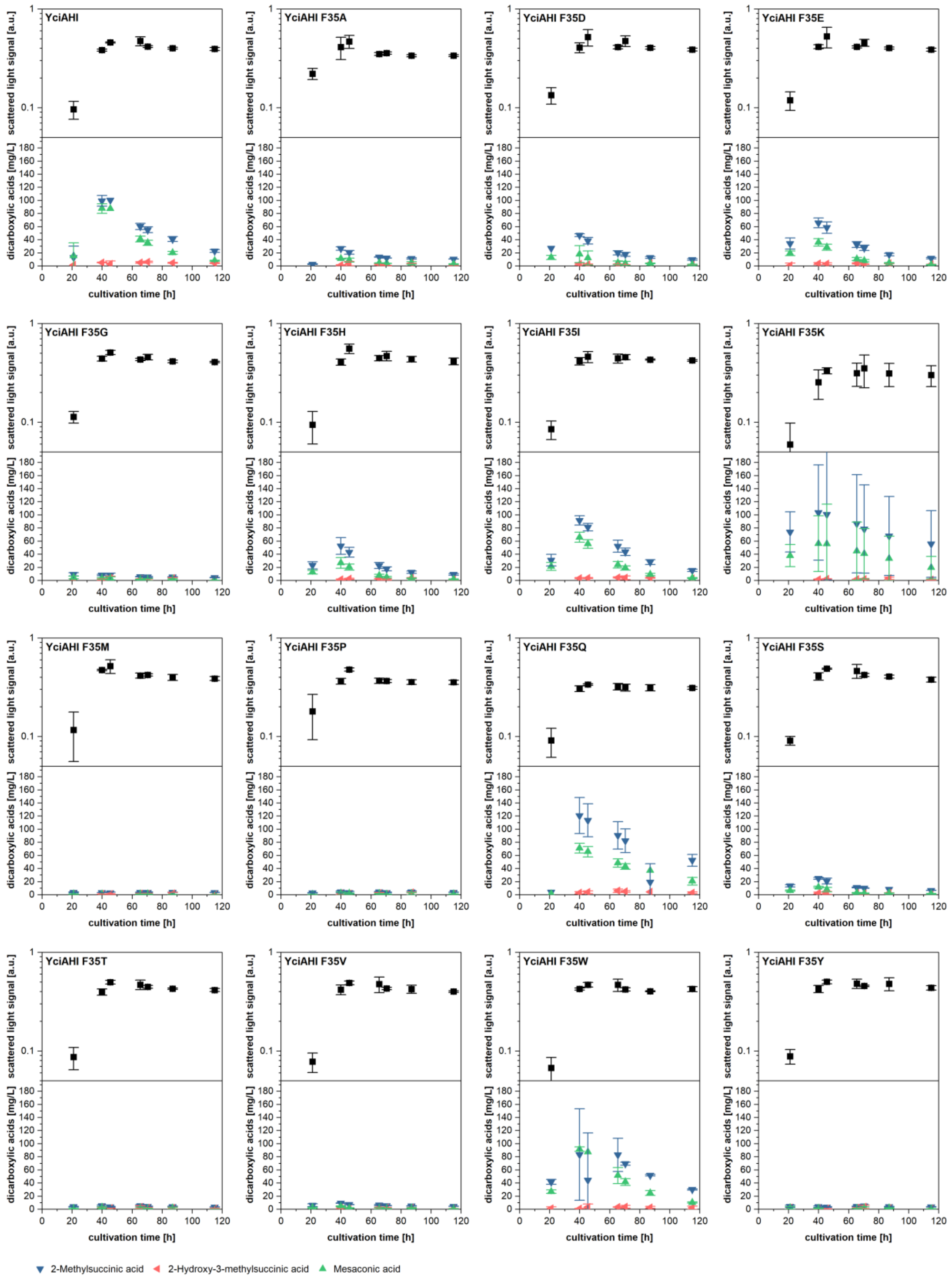

Fig. S4 Growth kinetics and time-dependent concentration of 2-methylsuccinic acid, 2-hydroxy-3-methylsuccinic acid and mesaconic acid in supernatant of *M. extorquens* AM1 harboring YciAHI F35 variants (untagged). EMCP-derived carboxylic acid products with titers insufficient for quantification (< 5 mg/L) are not displayed. Error bars represent standard deviations from three independent replicates

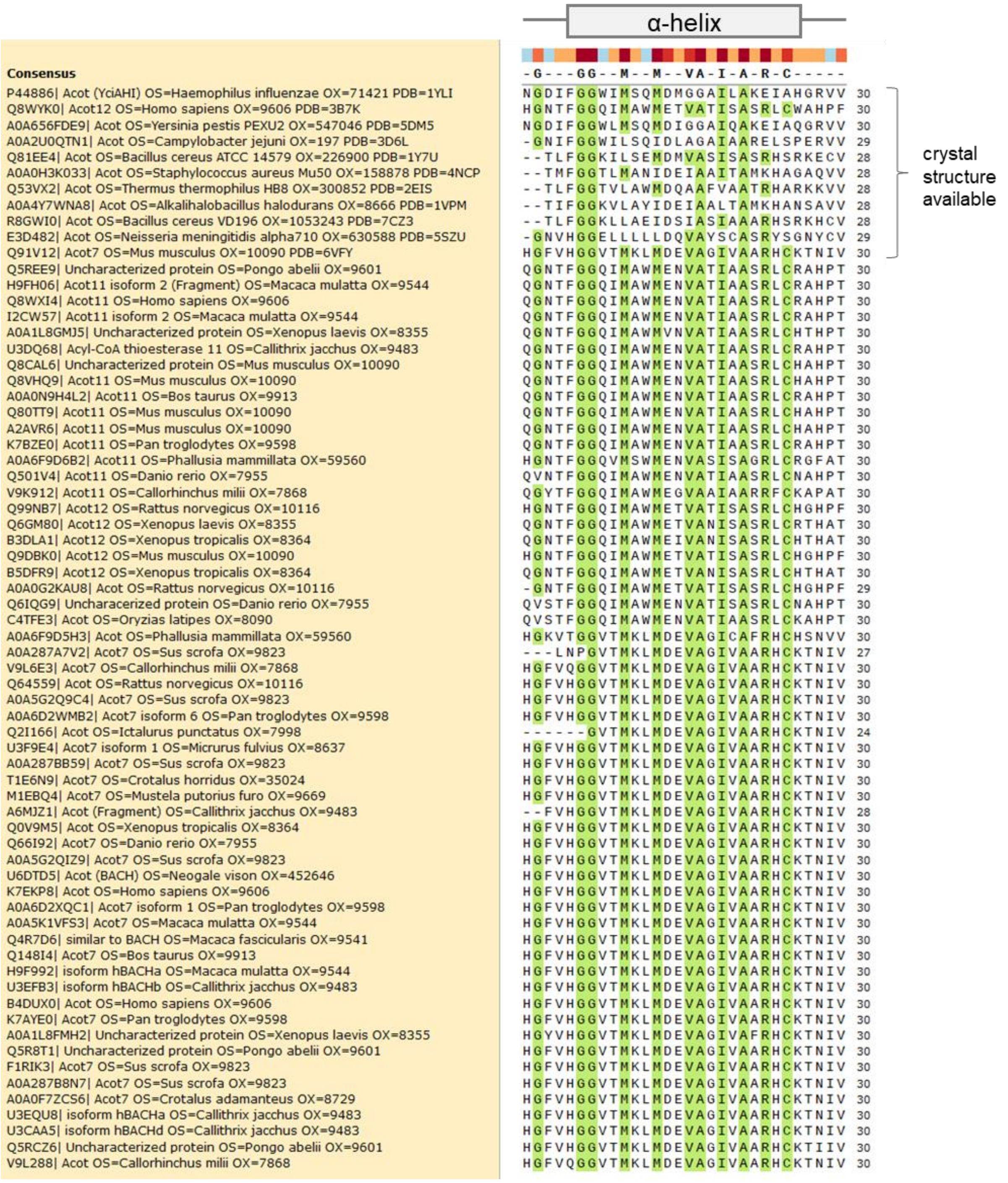

Fig. S5 Alignment of TE6 family members (α-helix domain). Sequences were acquired from ThYme (https://thyme.engr.unr.edu/v2.0/). Alignment was done in COBALT (Papadopoulos and Agarwala 2007) and visualized with SnapGene (GSL Biotech LLC, San Diego, Canada). Consensus with a threshold of 80% is shown. Amino acids corresponding to consensus are highlighted in green

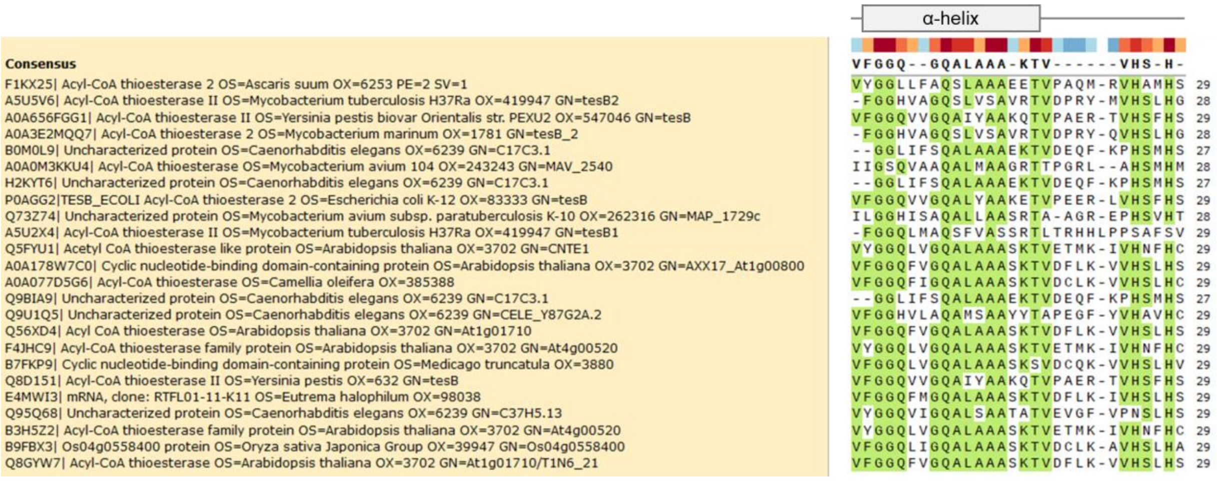

Fig. S6 Alignment of TE4 family members (α-helix domain). Sequences were acquired from ThYme (https://thyme.engr.unr.edu/v2.0/). Alignment was done in COBALT (Papadopoulos and Agarwala 2007) and visualized with SnapGene (GSL Biotech LLC, San Diego, Canada). Consensus with a threshold of 80% is shown. Amino acids corresponding to consensus are highlighted in green

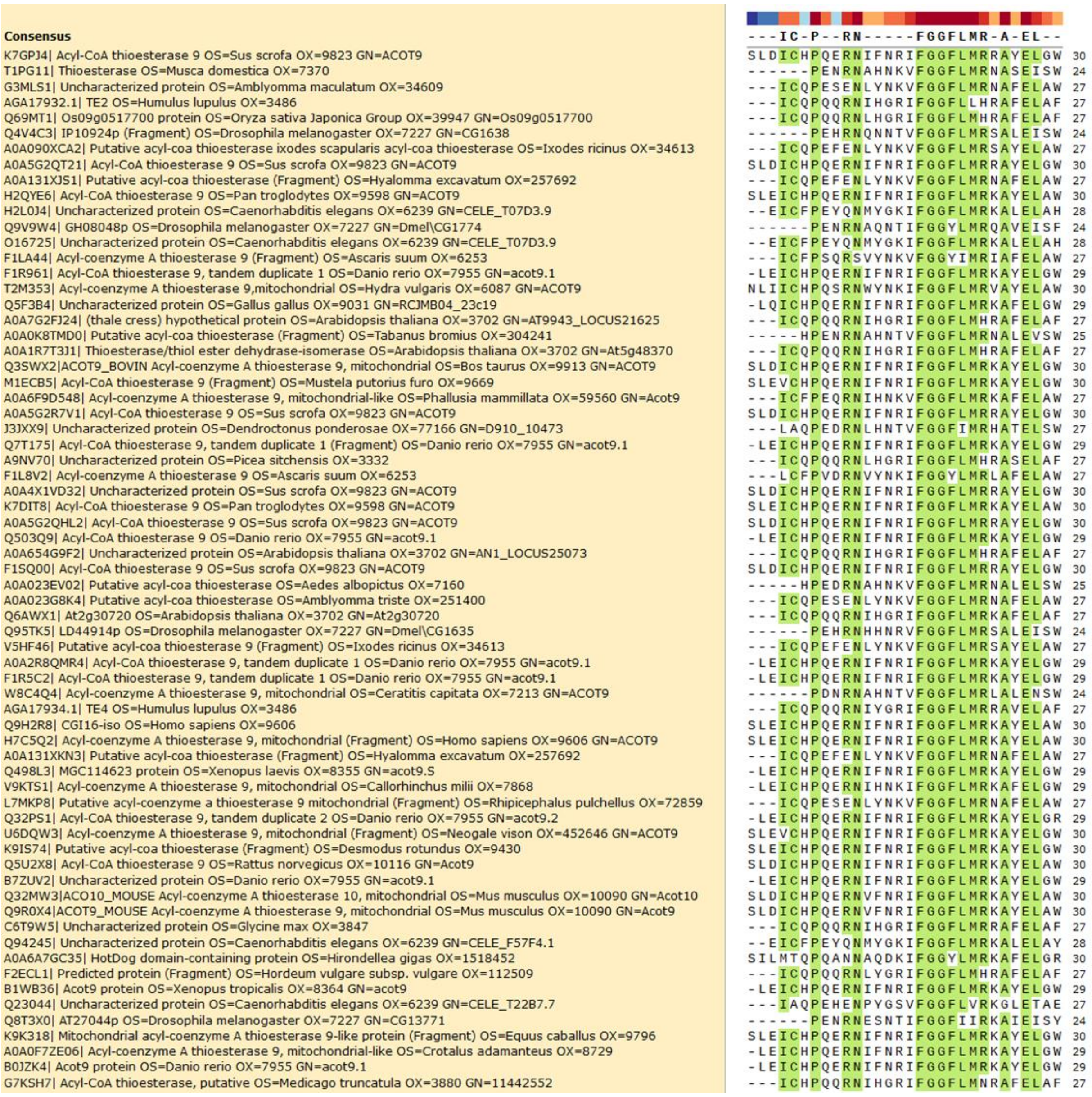

Fig. S7 Alignment of TE7 family members (presumed α-helix domain). Sequences were acquired from ThYme (https://thyme.engr.unr.edu/v2.0/). Alignment was done in COBALT (Papadopoulos and Agarwala 2007) and visualized with SnapGene (GSL Biotech LLC, San Diego, Canada). Consensus with a threshold of 80% is shown. Amino acids corresponding to consensus are highlighted in green

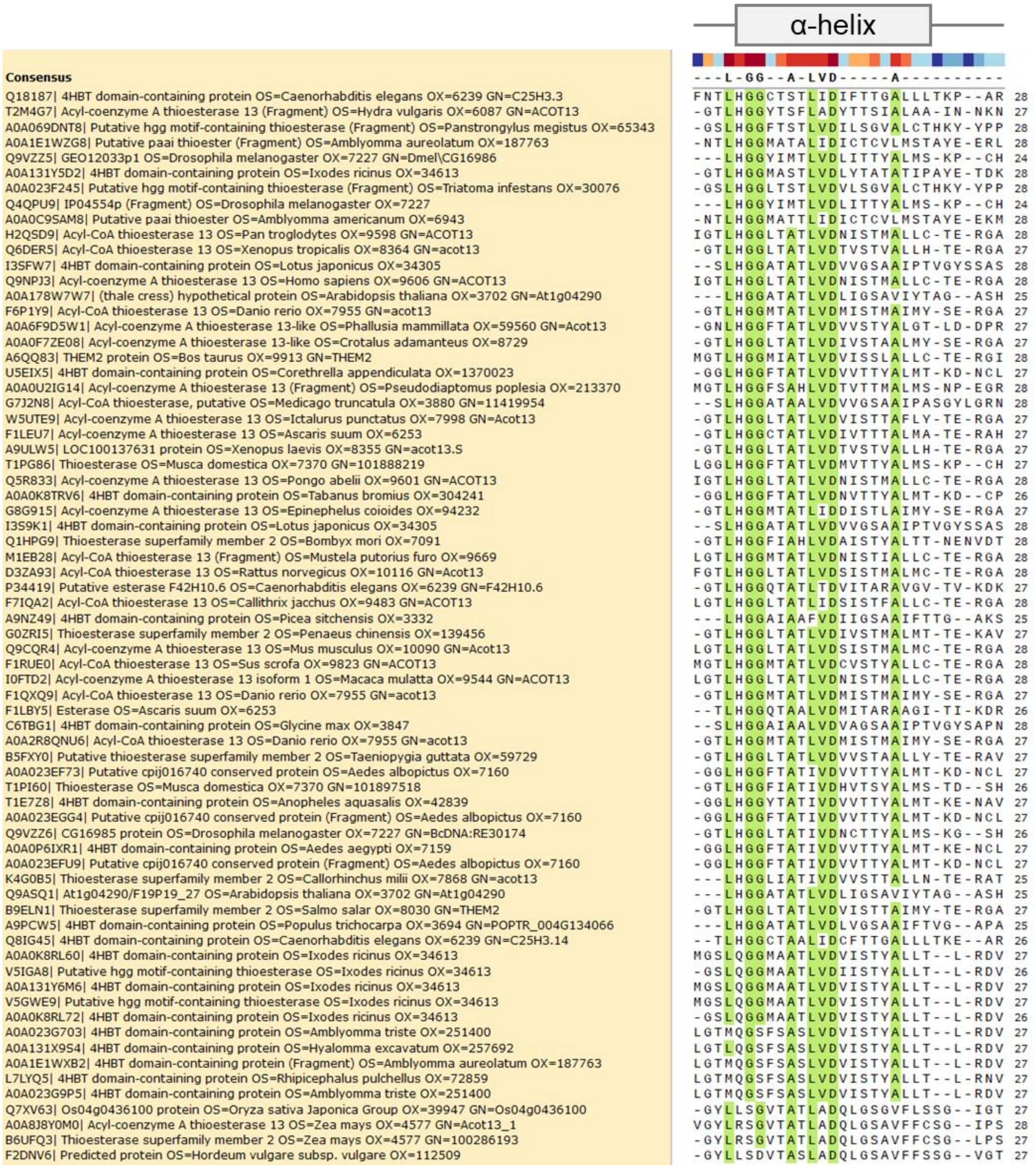

Fig. S8 Alignment of TE8 family members (α-helix domain). Sequences were acquired from ThYme (https://thyme.engr.unr.edu/v2.0/). Alignment was done in COBALT (Papadopoulos and Agarwala 2007) and visualized with SnapGene (GSL Biotech LLC, San Diego, Canada). Consensus with a threshold of 80% is shown. Amino acids corresponding to consensus are highlighted in green

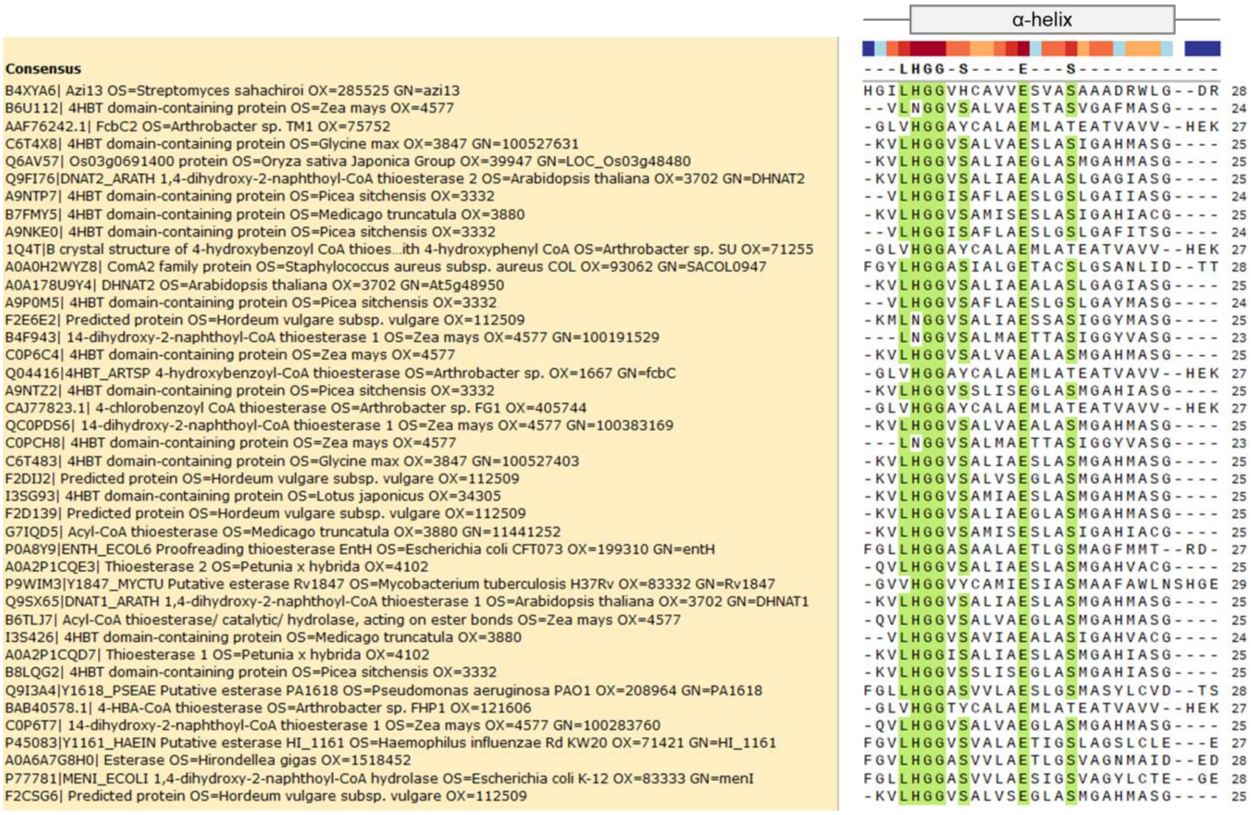

Fig. S9 Alignment of TE11 family members (α-helix domain). Sequences were acquired from ThYme (https://thyme.engr.unr.edu/v2.0/). Alignment was done in COBALT (Papadopoulos and Agarwala 2007) and visualized with SnapGene (GSL Biotech LLC, San Diego, Canada). Consensus with a threshold of 80% is shown. Amino acids corresponding to consensus are highlighted in green

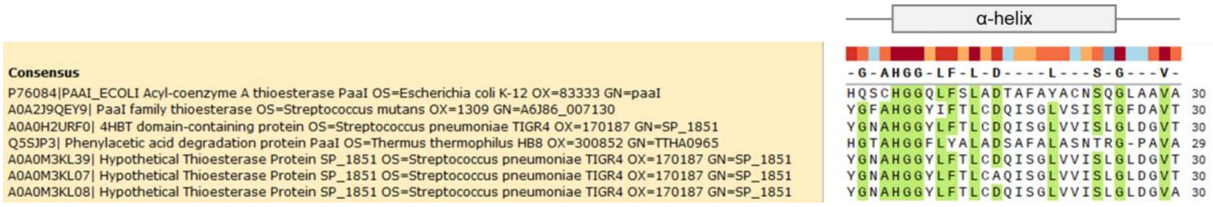

Fig. S10 Alignment of TE31 family members (α-helix domain). Sequences were acquired from ThYme (https://thyme.engr.unr.edu/v2.0/). Alignment was done in COBALT (Papadopoulos and Agarwala 2007) and visualized with SnapGene (GSL Biotech LLC, San Diego, Canada). Consensus with a threshold of 80% is shown. Amino acids corresponding to consensus are highlighted in green

Reference list

Papadopoulos JS, Agarwala R (2007) COBALT: constraint-based alignment tool for multiple protein sequences. Bioinformatics 23:1073–1079. https://doi.org/10.1093/bioinformatics/btm076
